# Targeting dendritic cells with TLR-2 ligand–coated nanoparticles loaded with *Mycobacterium tuberculosis* epitope induce antituberculosis immunity

**DOI:** 10.1016/j.jbc.2022.102596

**Published:** 2022-10-15

**Authors:** Deepjyoti Kumar Das, Mohammad Adeel Zafar, Sidhanta Nanda, Sanpreet Singh, Taruna Lamba, Hilal Bashir, Pargat Singh, Sudeep Kumar Maurya, Sajid Nadeem, Sharvan Sehrawat, Vijayender Bhalla, Javed Naim Agrewala

**Affiliations:** 1 Immunology Laboratory, CSIR-Institute of Microbial Technology, Chandigarh, India; 2 Department of Biomedical Engineering, Indian Institute of Technology Ropar, Rupnagar, India; 3 Department of Biological Sciences, Indian Institute of Science Education and Research, Mohali, India; 4 Biosensor Laboratory, CSIR-Institute of Microbial Technology, Chandigarh, India

**Keywords:** *Mycobacterium tuberculosis*, vaccine, nanotechnology, peptides, toll-like receptor-2, Ab, antibody, AFM, atomic force microscopy, APC, antigen-presenting cell, BCA, bicinchoninic acid, BCG, *Bacillus* Calmette–Guerin, bioP_3_, biotinylated P_3_, BSA, bovine serum albumin, CavME, caveolated-mediated endocytosis, CFSE, 5(6)-carboxyfluorescein diacetate *N*-succinimidyl ester, CME, clathrin-mediated endocytosis, DC, dendritic cell, DC^TLR-2−/−^, DC generated from TLR-2^−/−^, DC^WT^, DC generated from WT, DLS, dynamic light scattering, DTA, differential thermal analysis, EDC, *N*-(3-dimethylaminopropyl)-*N*′-ethylcarbodiimide hydrochloride, EE, entrapment efficiency, E_91_NP3-eFlour670, eFluor670 dye–labeled E_91_NP3, E_91_NP3-FITC, FITC-labeled E_91_NP3, FCS, fetal calf serum, FE-SEM, field emission scanning electron microscopy, FMT, fluorescence molecular tomography, GM-CSF, granulocyte–macrophage colony-stimulating factor, HLA, human leukocyte antigen, HRP, horseradish peroxidase, IFN-γ, interferon gamma, IL, interleukin, iNOS, inducible NO synthase, ITC, isothermal titration calorimetry, LN, lymph node, MHC, major histocompatibility complex, *Mtb*, *Mycobacterium tuberculosis*, NHS, *N*-hydroxylsuccinimide, NO, nitric oxide, NP, nanoparticle, PDI, polydispersity index, PI, propidium iodide, RT–qPCR, quantitative RT–PCR, SN, supernatant, TB, tuberculosis, TEM, transmission electron microscopy, TGA, thermogravimetric analysis, TLR-2, toll-like receptor-2, TNFα, tumor necrosis factor alpha, TPP, pentasodium tripolyphosphate hexahydrate, ζ-potential, zeta potential

## Abstract

Novel vaccination strategies are crucial to efficiently control tuberculosis, as proposed by the World Health Organization under its flagship program “End TB Strategy.” However, the emergence of drug-resistant strains of *Mycobacterium tuberculosis* (*Mtb*), particularly in those coinfected with HIV-AIDS, constitutes a major impediment to achieving this goal. We report here a novel vaccination strategy that involves synthesizing a formulation of an immunodominant peptide derived from the Acr1 protein of *Mtb*. This nanoformulation in addition displayed on the surface a toll-like receptor-2 ligand to offer to target dendritic cells (DCs). Our results showed an efficient uptake of such a concoction by DCs in a predominantly toll-like receptor-2–dependent pathway. These DCs produced elevated levels of nitric oxide, proinflammatory cytokines interleukin-6, interleukin-12, and tumor necrosis factor-α, and upregulated the surface expression of major histocompatibility complex class II molecules as well as costimulatory molecules such as CD80 and CD86. Animals injected with such a vaccine mounted a significantly higher response of effector and memory Th1 cells and Th17 cells. Furthermore, we noticed a reduction in the bacterial load in the lungs of animals challenged with aerosolized live *Mtb*. Therefore, our findings indicated that the described vaccine triggered protective anti-*Mtb* immunity to control the tuberculosis infection.

Currently, *Bacillus* Calmette–Guerin (BCG) is the only available vaccine against tuberculosis (TB) (1). A follow-up study conducted at Chengalpattu, India involving 281,161 individuals concluded that BCG offers limited protection against TB (2, 3). The protective efficacy of the BCG vaccine is highly variable (0% to 98%). Furthermore, it does not confer long-lasting immunity (4, 5). Interestingly, BCG is inefficient in protecting the major population of TB endemic regions (1, 6). Thus, the important attributes to be considered while designing a vaccine candidate for TB endemic regions are that the vaccine should not be neutralized by the pre-existing antibodies (Abs) against *Mycobacterium tuberculosis* (*Mtb*) antigens, consists of immunodominant epitopes, enhance the expression of major histocompatibility complex (MHC) and costimulatory molecules, and does not require extensive antigen processing (7). Therefore, a peptide vaccine with immunodominant T-cell epitopes may be considered suitable, as such a vaccine should not only have an immunodominant epitope in the formulation but also remove elements that could be potentially autoreactive or induce immunosuppression. Epitope-based vaccines are safer than live or attenuated vaccines since they do not include any organism (8). However, peptides are less immunogenic and restricted by the human leukocyte antigen (HLA) diversity. Some of these constraints can be adequately resolved by linking peptides to adjuvants and selecting promiscuous peptides (9, 10).

To overcome the aforementioned difficulties, we recently synthesized a self-adjuvanting peptide L91, which consists of an immunodominant epitope (sequence SEFAYGSFVRTVSLPVGADE, 91–110) of Acr1 protein of *Mtb* conjugated to Pam3Cys, a toll-like receptor-2 (TLR-2) ligand. L91 proved to be a promising vaccine candidate against TB because of its ability to generate enduring memory Th1 cells and Th17 cells and to confer to the host better protection than that achieved by BCG (11, 12).

Nanoparticle (NP)-based approaches can be used to formulate new generation vaccines. NP synthesis is a versatile process and requires less stringent purification processes and is easy to scale up. NPs can be suitably targeted to antigen-presenting cells (APCs), have good surface/volume ratios for efficient loading with the cargo, and are easy to modify. NPs can protect against the premature degradation of antigens, improve their stability, and help in targeting APCs for the effective delivery of vaccines (13). Chitosan is a cationic biopolymer, mucoadhesive, antibacterial, hypoallergic, biocompatible, and approved by the US Food and Drug Administration for drug delivery (14, 15). Chitosan triggers the cGAS-STING pathway to generate Th1-biased immunity, which is critical for protection against TB (16).

Pam3Cys, a ligand of TLR-2, is a potent adjuvant. TLR-2 signaling induces dendritic cell (DC) maturation and the elicitation of Th1 and Th17 immunity (17, 18, 19, 20). The chitosan NPs displaying DC-targeting peptides on their surface produce robust immunogenicity and remarkably improved antitumor immunity (21, 22). We, therefore, conjectured that delivering immunodominant *Mtb* epitopes through NPs to DCs would evoke a substantial immune response against the mycobacterium (23). We engineered peptide SEFAYGSFVRTVSLPVGADE (E_91_)-entrapped chitosan NPs expressing TLR-2 ligand Pam3Cys (P_3_) (E_91_NP_3_) on their surface. We observed that E_91_NP_3_ upregulated the expression of MHC II and costimulatory molecules on DCs, activated CD8 T cells, Th1 cells, and Th17 cells and induced elevated memory T-cell responses. Furthermore, animals immunized with the E_91_NP_3_ reduced the bacterial load in the lungs following *Mtb* exposure. We conclude that E_91_NP_3_ can induce remarkable immune responses to protect against TB.

## Results

### Synthesis and characterization of chitosan NPs

**Figure 1:**
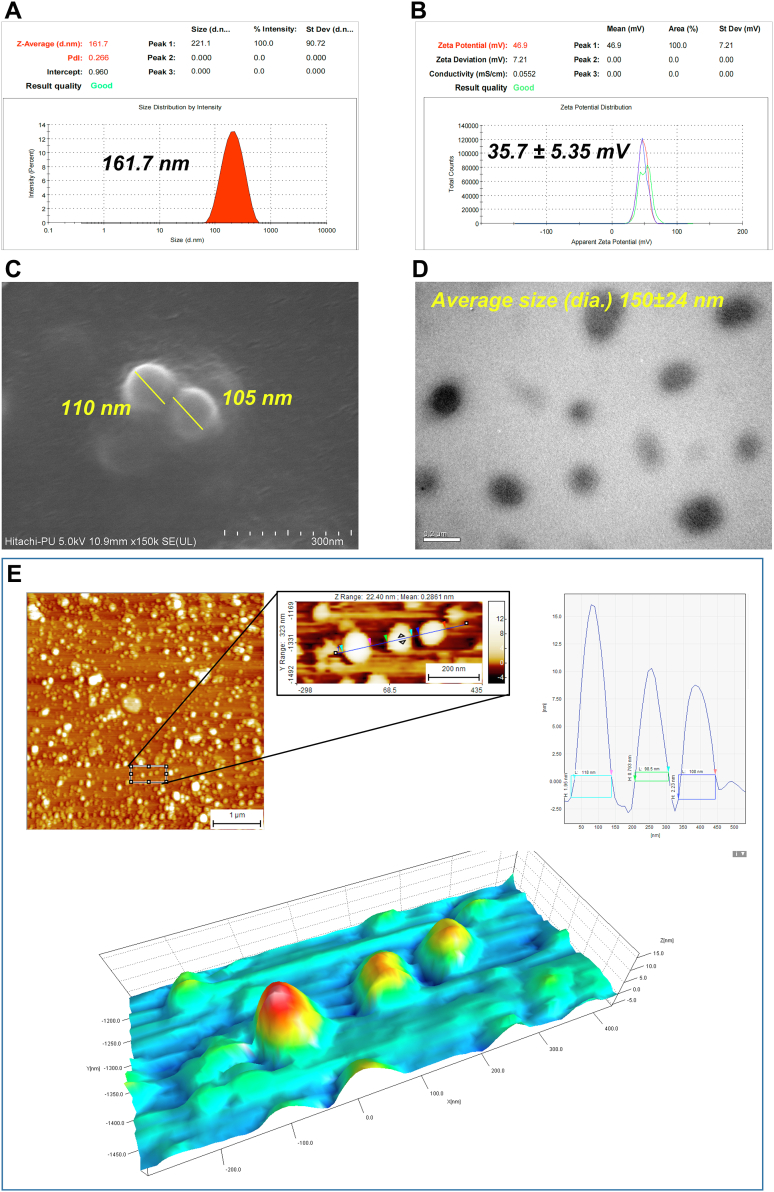
**Synthesis and characterization of chitosan nanoparticles (NPs).** Chitosan NPs were synthesized by the ionic-gelation method and used for characterization. *A*, DLS data depict the average hydrodynamic size and particle size distribution of the NPs. *B*, ζ-potential data of the NPs show the average surface charge of the NPs. *C*, FE-SEM and (*D*) TEM micrographs of the synthesized NPs represent the surface morphology and size, respectively. *E*, AFM micrograph, analyzed histogram, and 3D plot data depict the distribution and size analysis of well-dispersed NPs. The *arrows* in colors  in the histogram are corresponding to the colored *arrows* in the magnified image of the AFM micrograph. The *colored rectangles* show the point of contact in the respective histograms for size measurement for each NP. AFM, atomic force microscopy; DLS, dynamic light scattering; FE-SEM, field emission scanning electron microscopy; TEM, transmission electron microscopy; ζ-potential, zeta potential.

Chitosan NPs were synthesized by the ionic-gelation method and characterized by dynamic light scattering technique (DLS) (24). The NPs showed an average hydrodynamic diameter of ∼162 nm with a polydispersity index (PDI) value of 0.266 (Fig. 1*A*) and an average ζ-potential of +35.7 ± 5.35 mV (Fig. 1*B*). Furthermore, the morphology and size of NPs observed under field emission scanning electron microscopy (FE-SEM) were spherical with 105 to 110 nm diameter (Fig. 1*C*), and the transmission electron microscopy (TEM) micrograph depicts NP size of 150 ± 24 nm diameter (Fig. 1*D*). Furthermore, the atomic force microscopy (AFM) images and their analysis using SPIP software (Image Metrology) indicated NPs of average diameter ∼108 nm (Fig. 1*E*).

### Stability of NPs upon storage

The NPs were stable on storage at 4 °C and room temperature (22 ± 2 °C). The particle properties were listed in the DLS quality report for 1 month (Fig. S1*A*). Later, the NPs began to gradually swell, which occurred faster when kept at room temperature rather than 4 °C. On the other hand, freezing and thawing NPs had a significant impact on their size and stability. To avoid fast aggregation, cryoprotectants were utilized during the freezing process. We tested a variety of cryoprotectants and found that NPs were more stable in sucrose, d-glucose, and trehalose than in mannitol, PEG1000, and PEG3350 at −80 °C (Fig. S1*B*).

### Entrapment of E_91_ inside NPs (E_91_NP) and release profile of E_91_

**Figure 2:**
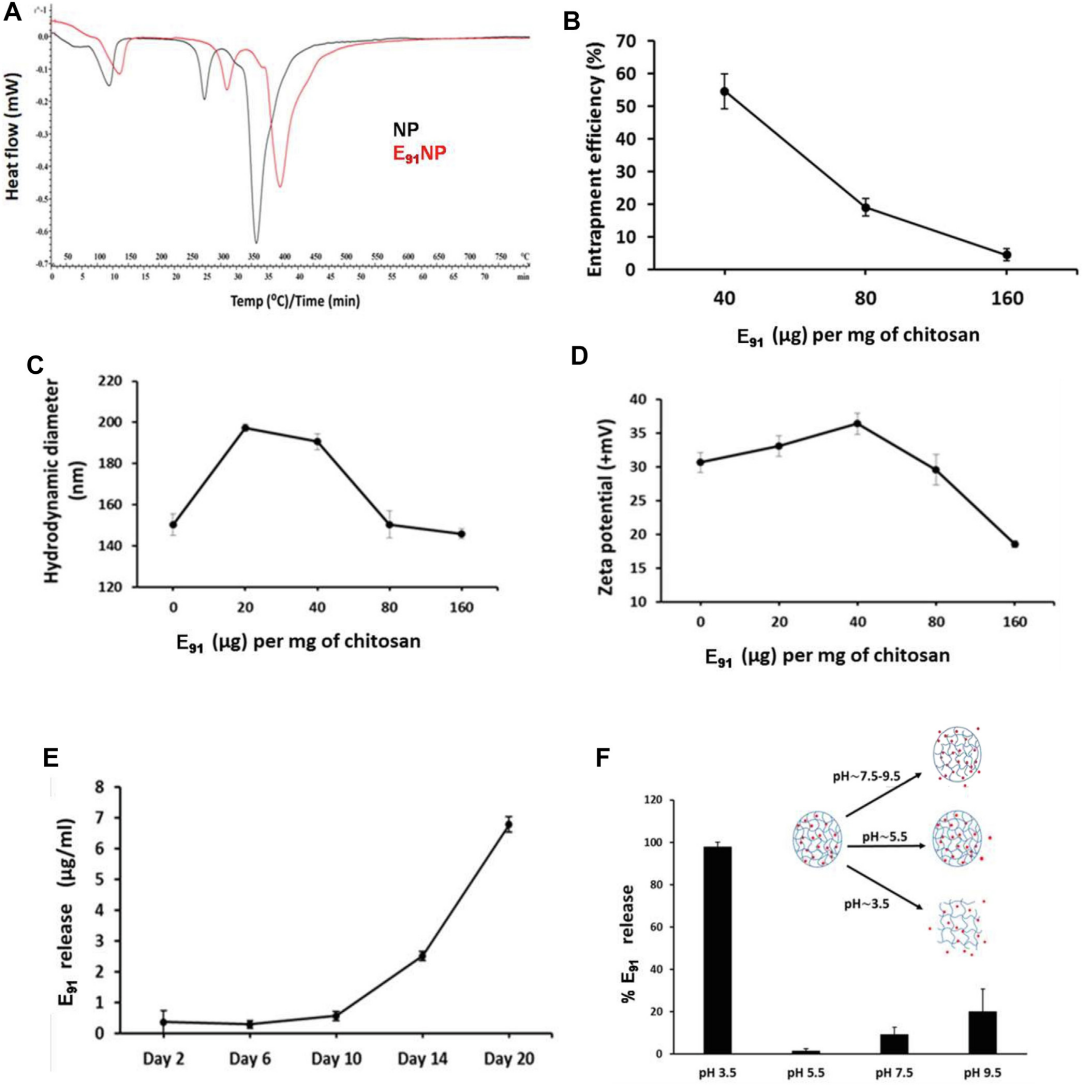
**Entrapment of E**_**91**_**inside nanoparticles (NPs) (E**_**91**_**NP) and release profile of E**_**91**_**.** E_91_ was entrapped inside the NPs during the synthesis process. *A*, the DTA graph compares the heat flow pattern between blank NPs and E_91_-entrapped NPs (E_91_NP). *B*–*D*, line charts show the changes in (*B*) entrapment efficiency, (*C*) DLS size, and (*D*) ζ-potential with an increasing amount of E_91_. E_91_ released along with time because of storage and burst release owing to change in pH is estimated using BCA. *E*, the line graph represents the time-dependent spontaneous release of entrapped peptides from the E_91_NPs. *F*, the bar graph demonstrates the burst release of E_91_ from E_91_NPs because of changes in pH. BCA, bicinchoninic acid; DLS, dynamic light scattering; DTA, differential thermal analysis; ζ-potential, zeta potential.

The immunodominant epitope (sequence: 91–110) of Acr1 of *Mtb* was used in the study. Its selection and protective efficacy have been established earlier (7, 11, 12). Differential thermal analysis (DTA) can quantify thermal stability because of changes in the chemical composition of the NPs (25). The DTA comparison between blank NPs and E_91_-entrapped NPs depicts an increase in the thermal stability of the NPs because of E_91_ entrapment (Fig. 2*A*). The estimation of E_91_ by bicinchoninic acid (BCA) showed that entrapment efficiency (EE; 55%) was highest in reactions involving 40 μg E_91_/mg chitosan (Fig. 2*B*). We observed that with the increasing concentration of E_91_ up to 40 μg/mg (E_91_/chitosan), there was an enhancement in both hydrodynamic sizes (Fig. 2*C*) as well as the ζ-potential (Fig. 2*D*). However, with the further addition of E_91_, both these parameters started decreasing. Thus, the E_91_: chitosan ratio of 40 μg : 1 mg giving a DLS size of ∼190 nm and ζ-potential of +35 mV was selected for all the subsequent experiments. Time-dependent gradual release of E_91_ peptide was observed from the NPs on storage at room temperature (Fig. 2*E*). The pH-dependent release of E_91_ from the NPs was checked at different pHs (3.5, 5.5, 7.5, and 9.5). A maximum release of the entrapped E_91_ peptides was observed at a pH of 3.5 (Fig. 2*F*). This might be due to a bursting of chitosan NPs at an acidic pH (26).

### Conjugation of Pam3Cys on the NPs surface

**Figure 3:**
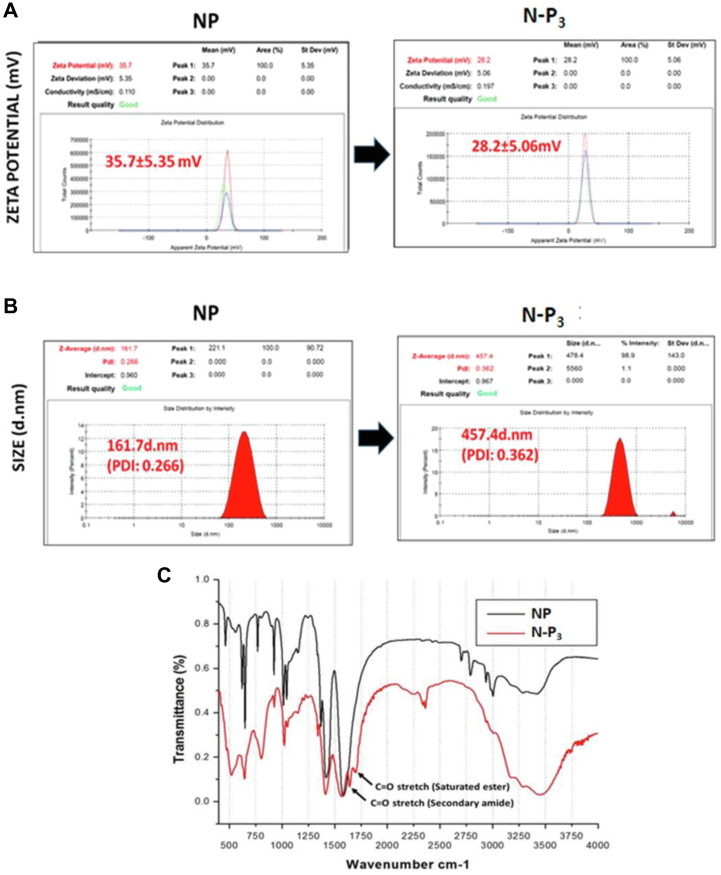

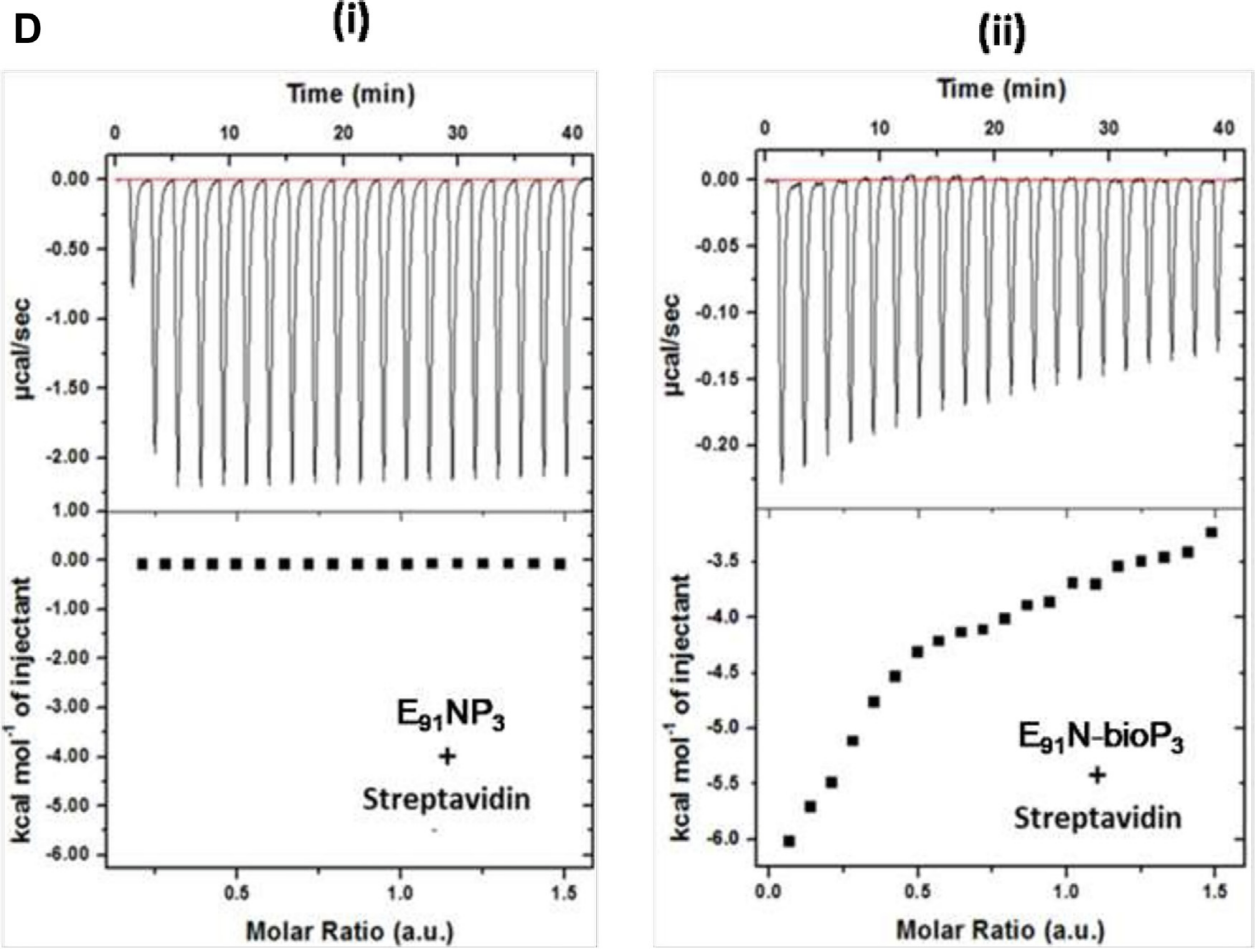
**Conjugation of P**_**3**_**on the surface of E**_**91**_**NP.** E_91_NPs were used to conjugate P_3_ on the surface using EDC–NHS chemistry. The detailed protocol for conjugation has been described in the Experimental procedures section. The conjugation was confirmed by several techniques. *A* and *B*, DLS plots show the changes in (*A*) ζ-potential (data compared with ζ-potential of blank NPs from Fig. 1*A*); (*B*) DLS size before and after P_3_ conjugation (data compared with a hydrodynamic diameter of blank NPs from Fig. 1*B*); (*C*) FTIR spectra showing the presence of C=O stretch of saturated ester (1742 cm^−1^) and 2° amide (1680 cm^−1^), imparted by the conjugation of P_3_ molecules on the surface of NPs. Biotinylated P_3_ (bio-P_3_) was used for isothermal titration calorimetry (ITC). *D*, heat flow profile of ITC exhibiting time upon injection of HRP–streptavidin to NPs surface-functionalized with either (i) P_3_ or bio-P_3_. DLS, dynamic light scattering; EDC, *N*-(3-dimethylaminopropyl)-*N*′-ethylcarbodiimide hydrochloride; HRP, horseradish peroxidase; NHS, *N*-hydroxylsuccinimide; NP, nanoparticle; ζ-potential, zeta potential.

Pam3Cys (P_3_), a ligand of TLR-2, was conjugated on the surface of E_91_NP to generate E_91_NP-P_3_ (E_91_NP_3_). After the conjugation of P_3_ on the surface of NPs, ζ-potential decreased from +35.7 ± 5.35 mV to +28.2 ± 5.06 mV (Fig. 3*A*), whereas the hydrodynamic size increased from ∼160 nm (PDI = 0.266) to ∼460 nm (PDI = 0.362) (Fig. 3*B*). TEM images of E_91_NP_3_ confirmed this increase in the size of NPs because of the conjugation of P_3_ (Fig. S2*A*). Similarly, an increase in the size because of lipid conjugation on chitosan NPs has been observed. The FTIR spectroscopy profile for saturated ester (1742 cm^−1^) and 2° amide (1680 cm^−1^) displayed unique signature peaks of C=O stretch, indicating effective conjugation of P3 onto NPs surface (27, 28) (Fig. 3*C*). Furthermore, ELISA results confirmed that biotinylated-P_3_ was successfully conjugated on the surface of E_91_NP (Fig. S2*B*). Furthermore, we conducted isothermal titration calorimetry (ITC) to confirm the conjugation of P_3_ on the NP surface. Our results showed an interaction between E_91_NP-biotinylated-P_3_ and horseradish peroxidase (HRP)–streptavidin (Fig. 3*D*). The ITC experiments corroborated our results for P_3_ conjugation on the E_91_NP surface. The E_91_NP_3_ was stable for 60 days at 4 °C, since no leakage of the E_91_ was noticed (Fig. S2*C*).

### E_91_NP3-activated DCs secreted nitric oxide, interleukin-6, interleukin-12, and tumor necrosis factor alpha and upregulated the expression of costimulatory and MHC II molecules

**Figure 4:**
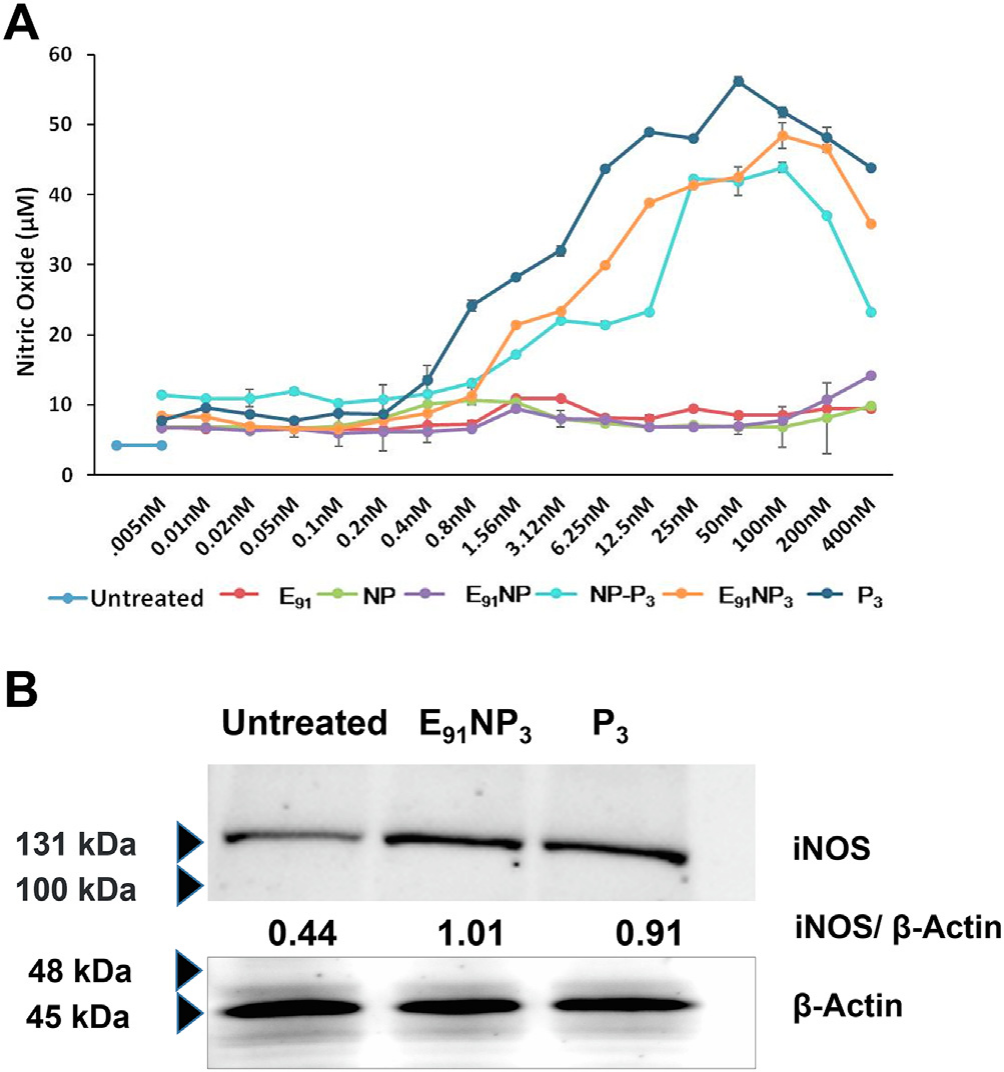

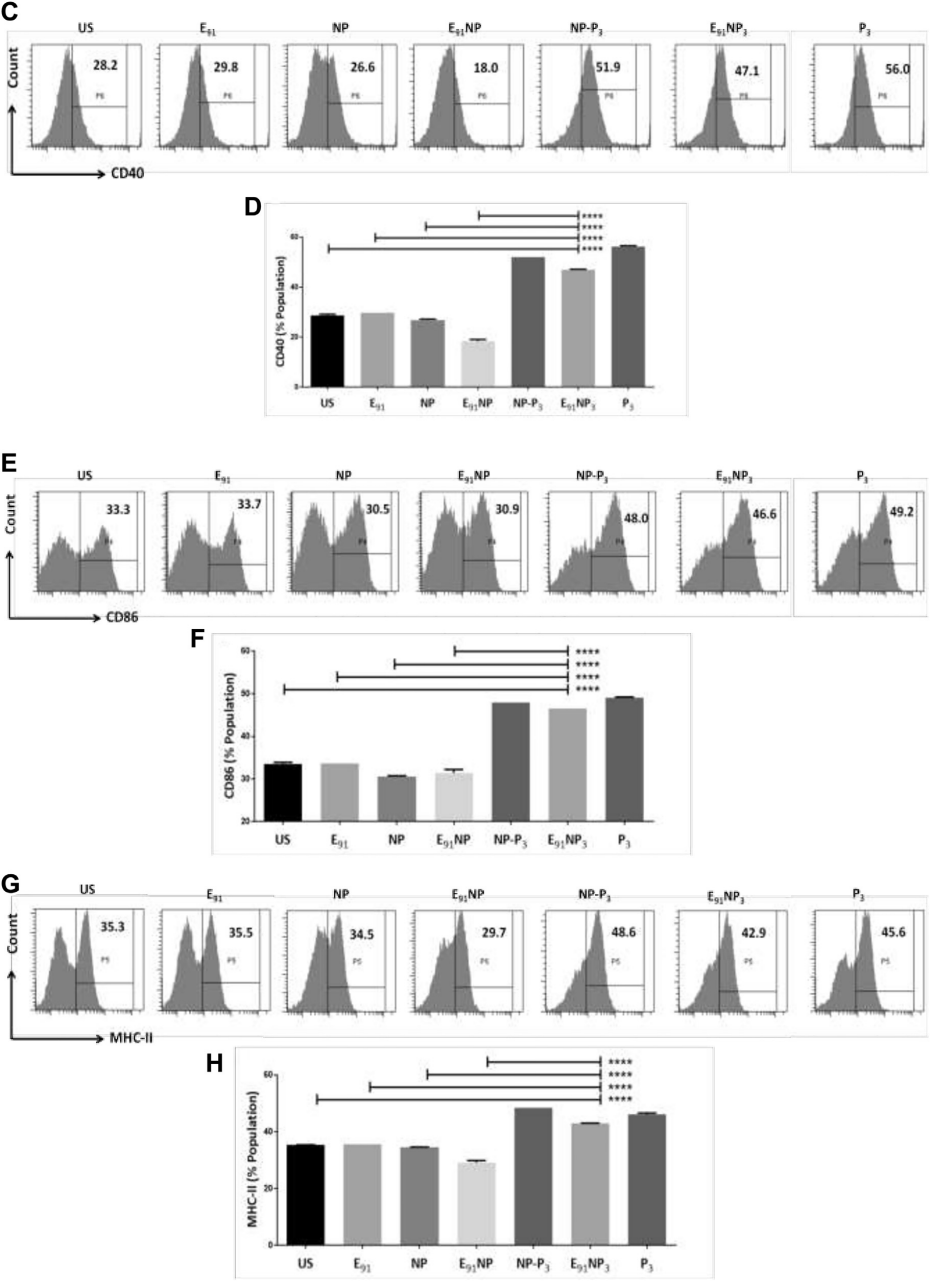

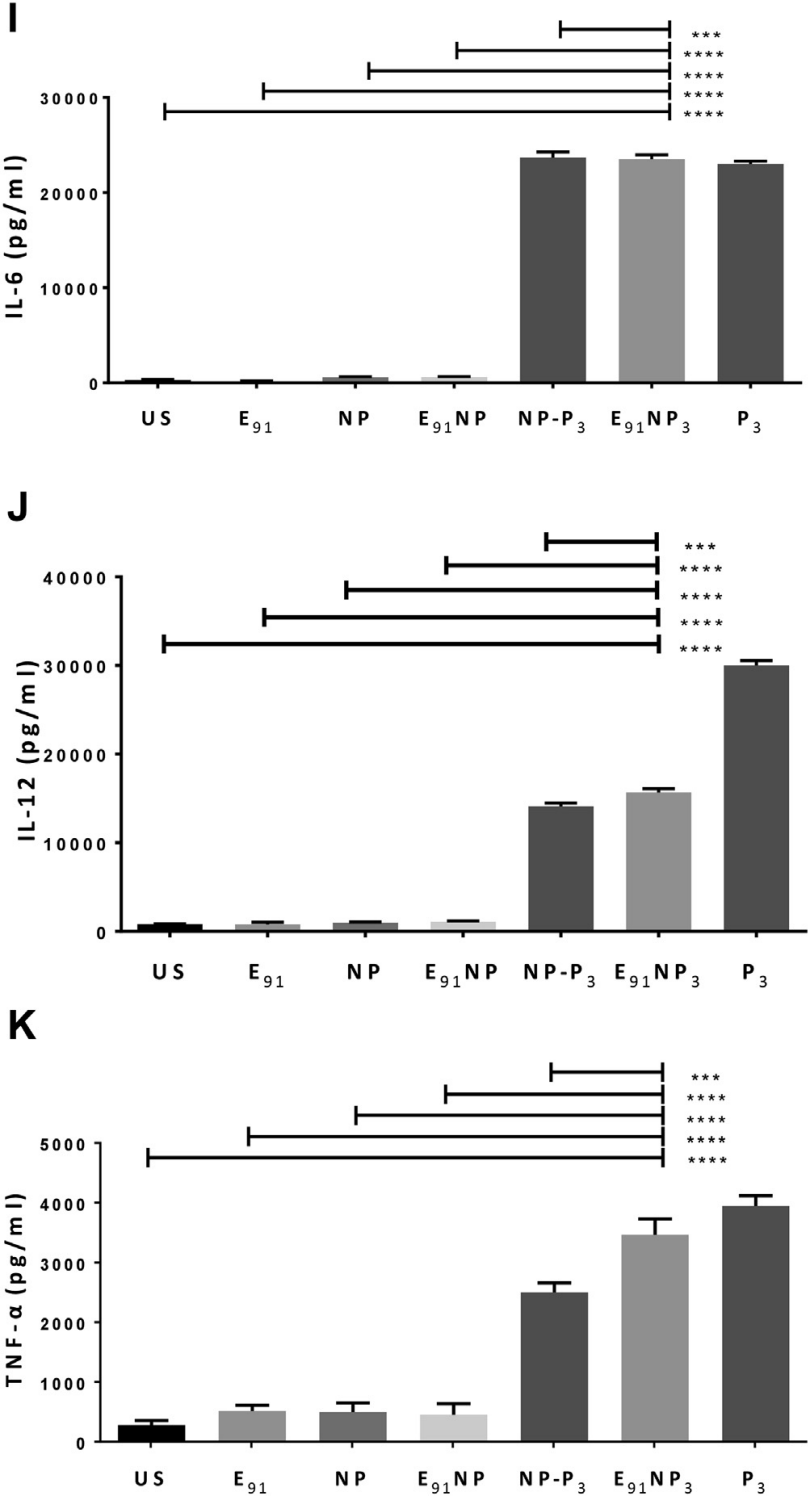
**E**_**91**_**NP**_**3**_**induces robust activationofDCs.** The DCs were cultured with E_91_NP_3_. Control cultures were set using E_91_, NP, E_91_NP, NP-P_3_, and P_3_. After 24 h, the cells were used for the expression of CD40, CD86, and MHC II by flow cytometry and SNs for the estimation of NO, IL-6, IL-12, and TNF-α production. *A*, line plot depicts the dose-dependent release of NO; (*B*) Western blot experiment was performed using the lysate prepared of the cells harvested at 5 to 6 h post-treatment. Blot depicts the expression levels of iNOS protein; β-actin was used as a loading control. The surface expression of (*C* and *D*) CD40; (*E* and *F*) CD86; (*G* and *H*) MHC II molecules was done by flow cytometry. *D*, *F*, and *H*, the flow cytometry data have also been expressed as bar diagrams. *I*–*K*, bar diagrams indicate the level (nanogram per milliliter) of (*I*) IL-6, (*J*) IL-12, and (*K*) TNFα estimated by ELISA. The data depicted as mean ± SD are representative of two to three independent experiments. ∗∗∗∗*p* < 0.0001. DC, dendritic cell; IL, interleukin; iNOS, inducible NO synthase; MHC, major histocompatibility complex; NO, nitric oxide; NP, nanoparticle; SN, supernatant; TNF-α, tumor necrosis factor alpha.

Nitric oxide (NO) helps in the activation of DCs by inhibiting caspase-like activity (29). We treated DCs with E_91_NP_3_ in the concentration ranges of 0.05 nM to 4000 nM and observed a dose-dependent increase in the secretion of NO. The maximum release of NO was noted with 50 nM of E_91_NP_3_ (Fig. 4*A*). Similar results were seen in the case of control NP-P_3_. No change was observed in the control cultures of E_91_NP, E_91_, and NP. This supports the stimulatory function of Pam3Cys (P_3_) in the E_91_NP_3_ and NP-P_3_ in successfully stimulating DCs. These results were corroborated by Western blotting experiments, which showed an augmented expression of inducible NO synthase (iNOS), the protein responsible for NO secretion (Fig. 4*B*).

The hallmark of activated DCs is the upregulation of the expression of MHC molecules and costimulatory molecules and the secretion of proinflammatory cytokines (30). The flow cytometry data revealed that DCs treated with E_91_NP_3_ exhibited higher expression of CD40, CD86, and MHC II molecules, as compared with the untreated cells, and these observations also supported the surface display of Pam3Cys in the E_91_NP_3_ formulation (Fig. 4, *C*–*H*). The culture supernatants (SNs) of DCs added with E_91_NP_3_ demonstrated elevated levels of proinflammatory cytokines, *viz*. interleukin 6 (IL-6), IL-12, and tumor necrosis factor alpha (TNF-α) (Fig. 4, *I*–*K*). These cytokines are crucial for protection against various pathogens, including *Mtb* (31, 32, 33). That the formation was not toxic to cells was shown by measuring the viability of DCs and as well as kidney cells (human embryonic kidney-293 cells) across a range of concentrations of E_91_NP_3_ added to the cultured cells, as measured by propidium staining (Fig. S3, *A* and *B*).

### E_91_NP_3_ activates DCs through the TLR-2 pathway

**Figure 5:**
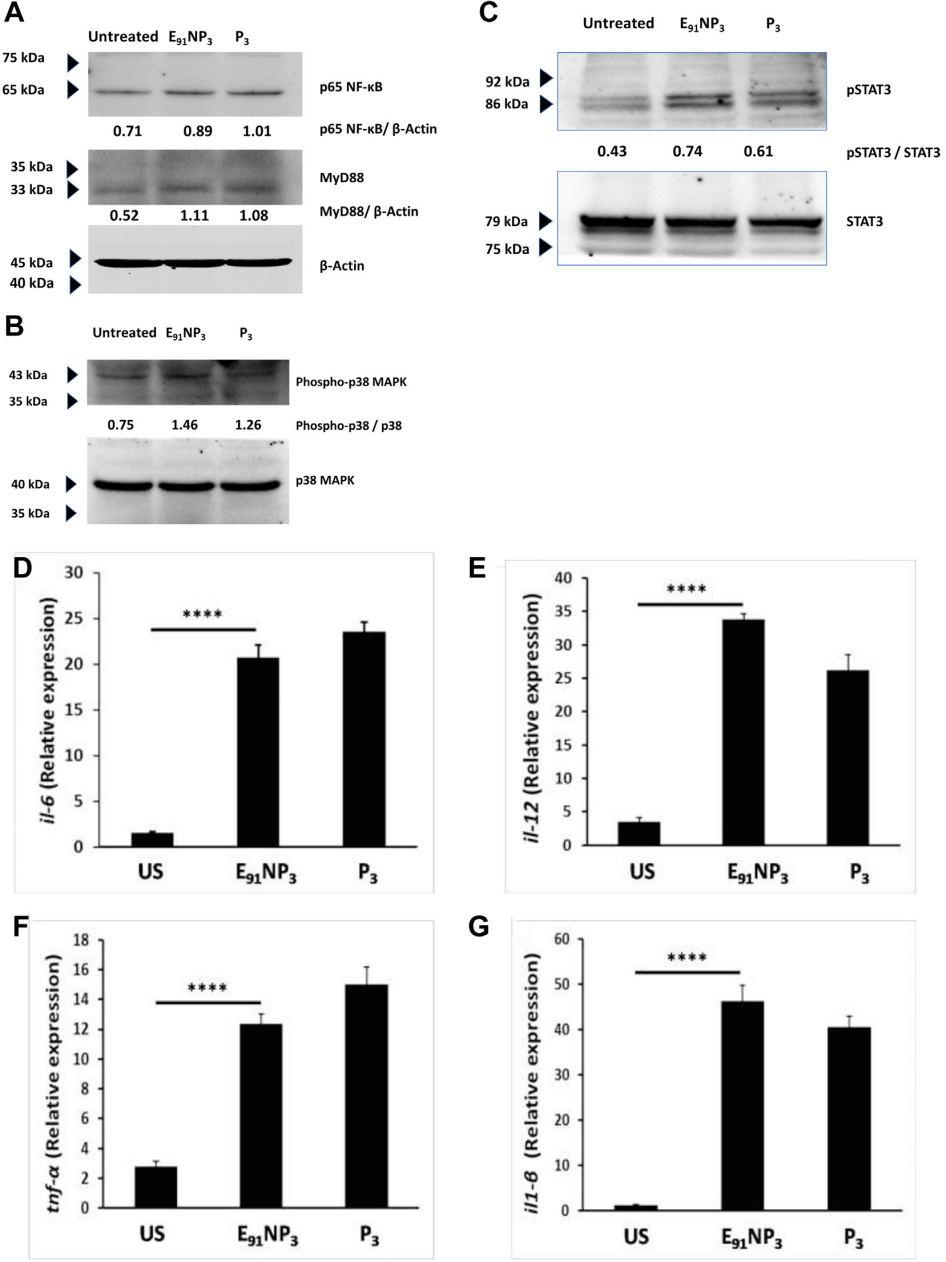

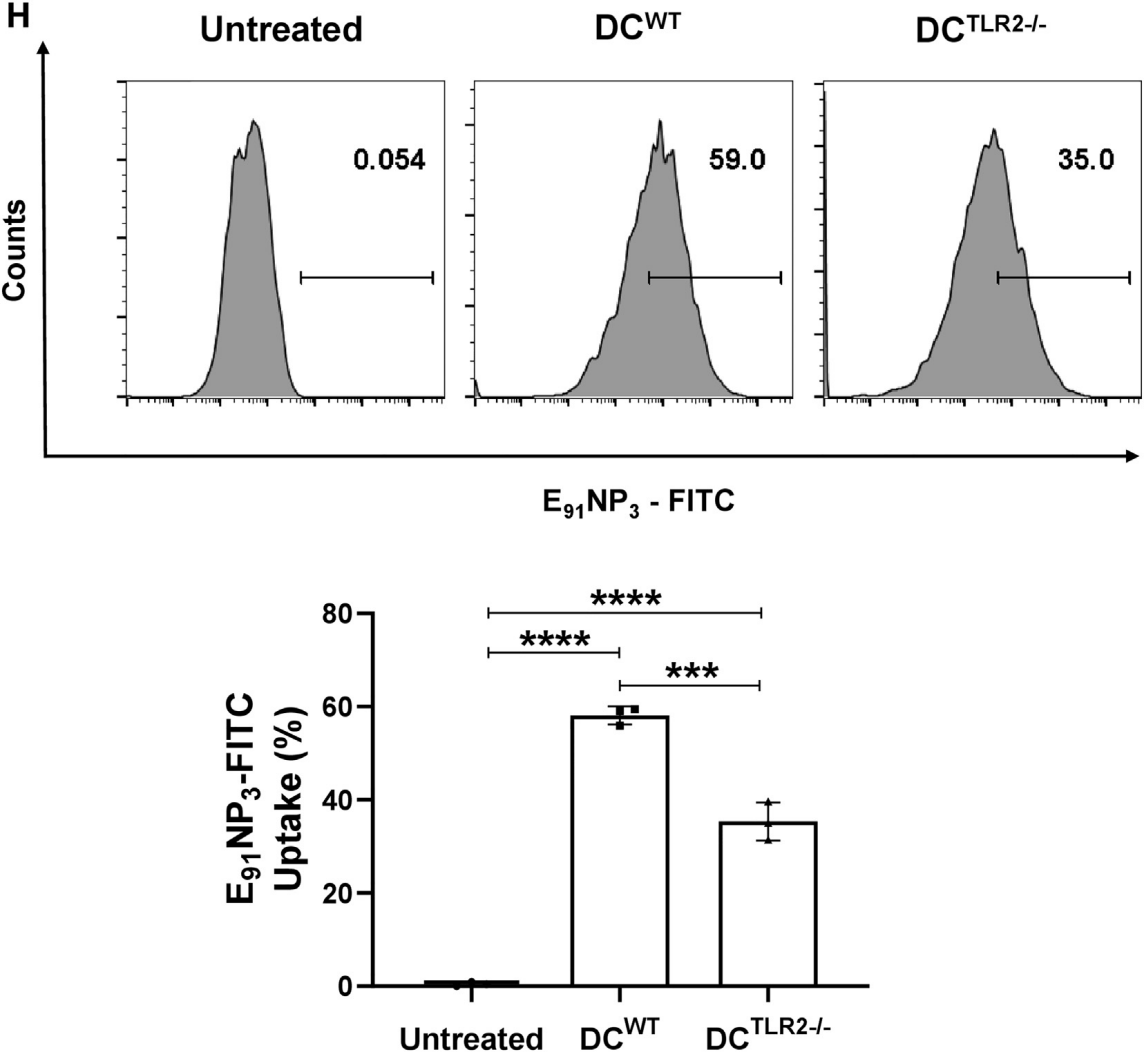

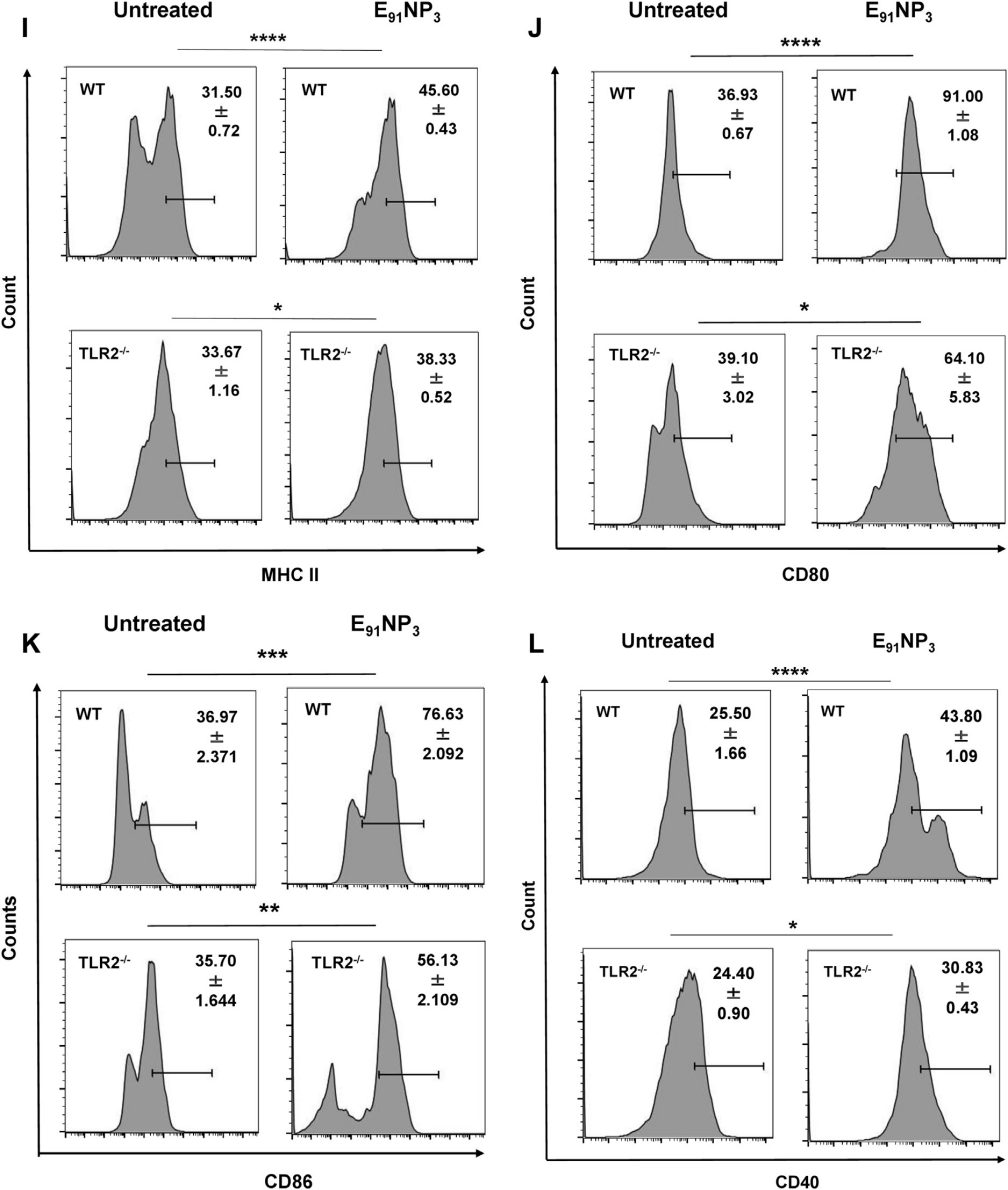
**E**_**91**_**NP**_**3**_**activates DCs through the TLR-2 pathway.** The DCs were cultured with E_91_NP_3_ and controls (P_3_, unstimulated DCs). After 5 to 6 h of stimulation, the cell lysate was prepared, and Western blotting was done. Blots depict the level of the expression of (*A*) p65 (NF-κB) and MyD88, (*B*) p38 and phospho-p38, and (*C*) STAT3 and phospho-STAT3 proteins. To check the gene level expression of different cytokines, qRT–PCR was performed using cDNA prepared from RNA isolated from the E_91_NP_3_-stimulated DCs and controls. *D*–*G*, bar graphs depict the relative expression of (*D*) *il6*, (*E*) *il12*, (*F*) *tnfα*, and (*G*) *il1β* genes. *H*–*K*, the DCs generated from WT (DC^WT^) and TLR-2^−/−^ (DC^TLR-2−/−^) C57BL/6 mice were cultured with FITC-labeled-E_91_NP_3_ for 4 h. Later, the cells were monitored for the (*H*) uptake of FITC-labeled-E_91_NP_3_ by the DC^WT^ and DC^TLR2^^−/−^; (*I*–*L*) expression of MHC II, CD80, CD86, and CD40 by the flow cytometer. The data (mean ± SD) shown are representative of two to three independent experiments. ∗*p* < 0.05, ∗∗*p* < 0.01, ∗∗∗*p* < 0.001, and ∗∗∗∗*p* < 0.0001. cDNA, complementary DNA; DC, dendritic cell; MHC, major histocompatibility complex; NP, nanoparticle; qRT–PCR, quantitative RT–PCR; TLR-2, toll-like receptor-2.

DCs highly express TLR-2 on their surface. Pam3Cys is a ligand of TLR-2 and thus activates such cells by engaging TLR-2 (34). Hence, we further substantiated the functionality of Pam3Cys in E_91_NP_3_ by monitoring the TLR-2 signaling pathway. The DCs were stimulated with E_91_NP_3_ and monitored for the expression levels of MyD88 and p65 of NF-κB pathways. E_91_NP_3_-stimulated DCs exhibited substantially higher expression of MyD88 and p65 NF-κB, as compared with unstimulated DCs (Fig. 5*A*). Furthermore, increased phosphorylation of p38 and STAT3 was observed in the E_91_NP_3_ pulsed DCs (Fig. 5, *B* and *C*). We also observed higher expression of proinflammatory cytokines, such as *il6*, *il12*, *tnfα*, and *il1β* by quantitative RT–PCR (RT–qPCR) (Fig. 5, *D*–*G*). Similar results were observed with the control DCs cultured with Pam3Cys. Thus, validating the Pam3Cys-induced events in the pulsed cells.

Since E_91_NP_3_ displays TLR-2 ligand Pam3Cys on its surface, we were curious to know whether it ligates TLR-2 and is endocytosed using the TLR2-dependent pathway. The DCs generated from TLR-2^−/−^ (DC^TLR-2−/−^) and WT (DC^WT^) mice were cultured with FITC-labeled E_91_NP_3_ (E_91_NP_3_-FITC). The significantly greater uptake of E_91_NP_3_-FITC was noticed in DC^WT^ (*p* < 0.001), compared with the DC^TLR-2−/−^. However, as compared with untreated DCs, both DC^WT^ and DC^TLR-2−/−^ showed significantly higher (*p* < 0.0001) uptake of the E_91_NP_3_-FITC (Fig. 5*H*). DC^WT^ showed considerably higher expression of MHC II, CD80, CD86, and CD40 than the DC^TLR-2−/−^ (Fig. 5, *I*–*L*). These results suggest that E_91_NP_3_ is predominantly endocytosed through the TLR-2-dependent pathway, and comparatively to a lesser extent by the non-TLR-2 pathway, resulting in the activation of both DCs and DC^TLR-2−/−^.

### Mechanism of E_91_NP_3_ uptake by DCs

**Figure 6:**
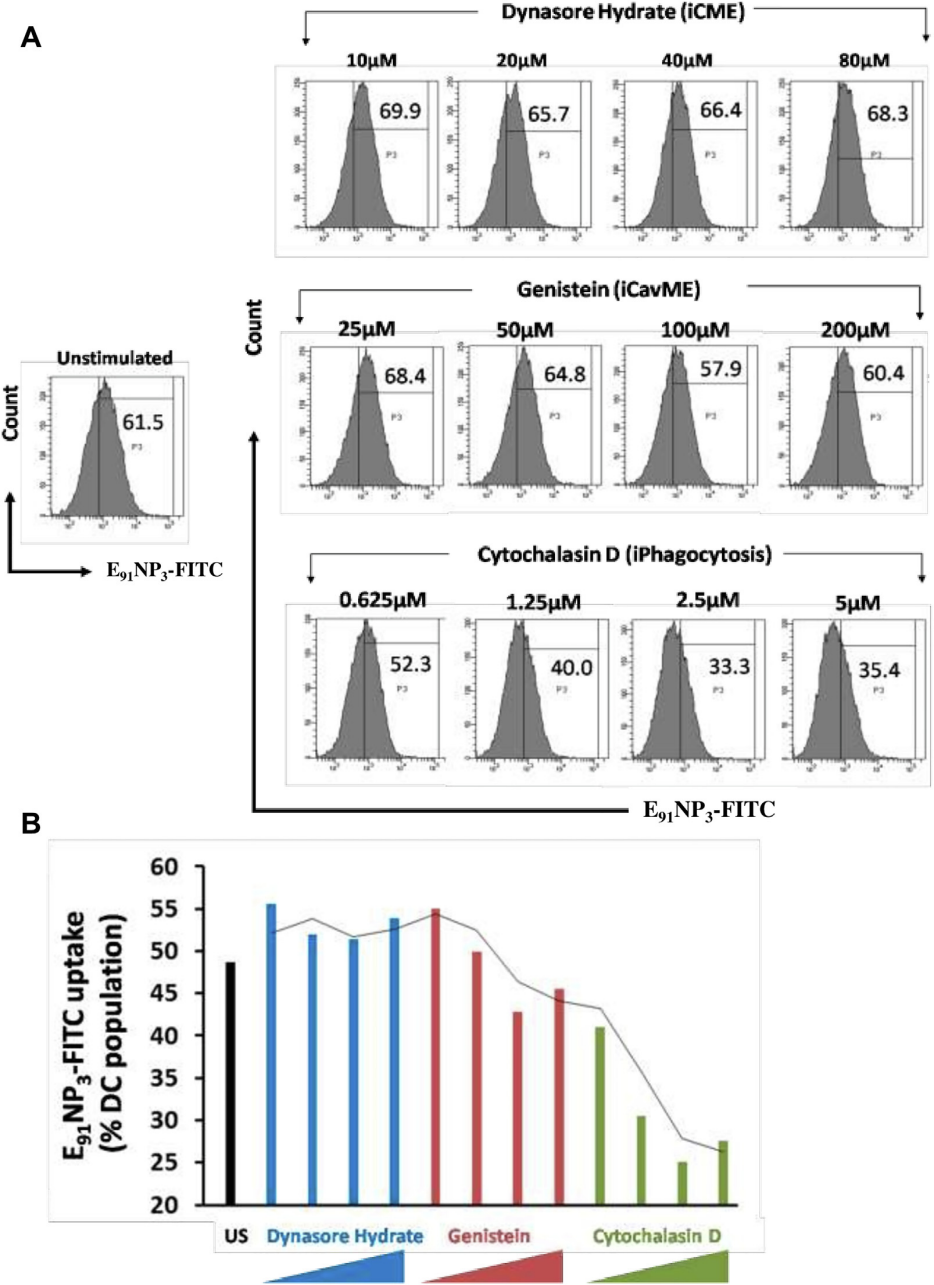
**Mechanism of E**_**91**_**NP**_**3**_**uptake by DCs.** The DCs were preincubated with inhibitors of dynasore hydrate (10–80 μM), genistein (25–200 μM), or cytochalasin D (0.625–5 μM) for 3 h. Later, DCs were incubated with E_91_NP_3_-FITC or medium alone (control) for 24 h. The DCs were harvested and stained for flow cytometry. *A*, the histogram and (*B*) bar graph show the uptake of E_91_NP_3_-FITC by DCs. Histograms are representative of two independent experiments, and the bar graph denotes the mean of two different experiments. The *greyline* in the bar diagram signifies the drift in E_91_NP_3_-FITC uptake with the increasing doses of the concerned inhibitors. DC, dendritic cell; NP, nanoparticle.

**Supplemental Figure 4 A-C:**
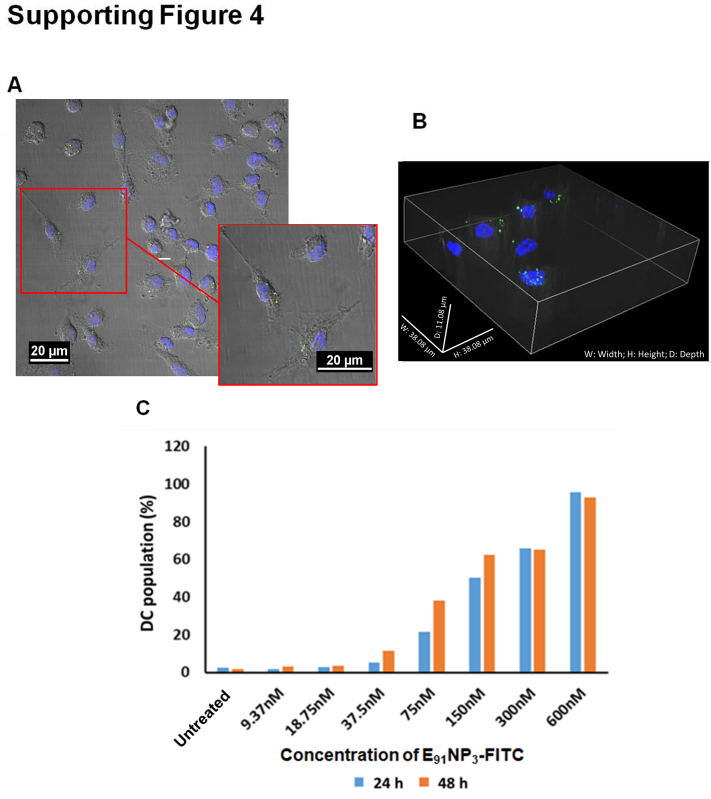


For the ingestion of foreign solid particles, mammalian cells use clathrin and caveolin-mediated endocytosis (35). Different intermediaries and mechanisms are utilized by these pathways. As a result, many pharmacologic inhibitors are available to stop these endocytic processes. Clathrin-mediated endocytosis (CME; inhibitor of CME), caveolated-mediated endocytosis (CavME; inhibitor of CavME), and phagocytosis can be chemically inhibited by dynasore hydrate, genistein, and cytochalasin D, respectively. We investigated the mechanism of E_91_NP_3_ uptake by DCs cultured for 24 and 48 h with E_91_NP_3_-FITC. The data from confocal imaging and flow cytometry revealed that DCs took up a significant amount of E_91_NP_3_-FITC (Fig. S4, *A*–*C*). DCs pretreated with escalating dosages of these inhibitors prior to incubating with E_91_NP_3_-FITC for 24 h showed that cytochalasin D but not dynasore hydrate and genistein inhibited the uptake process (Fig. 6, *A* and *B*). This indicated that E_91_NP_3_-FITC uptake was mediated by phagocytosis and not by clathrin or the caveolin-mediated pathways.

### Intracellular trafficking of E_91_NP_3_ in the DCs

**Figure 7:**
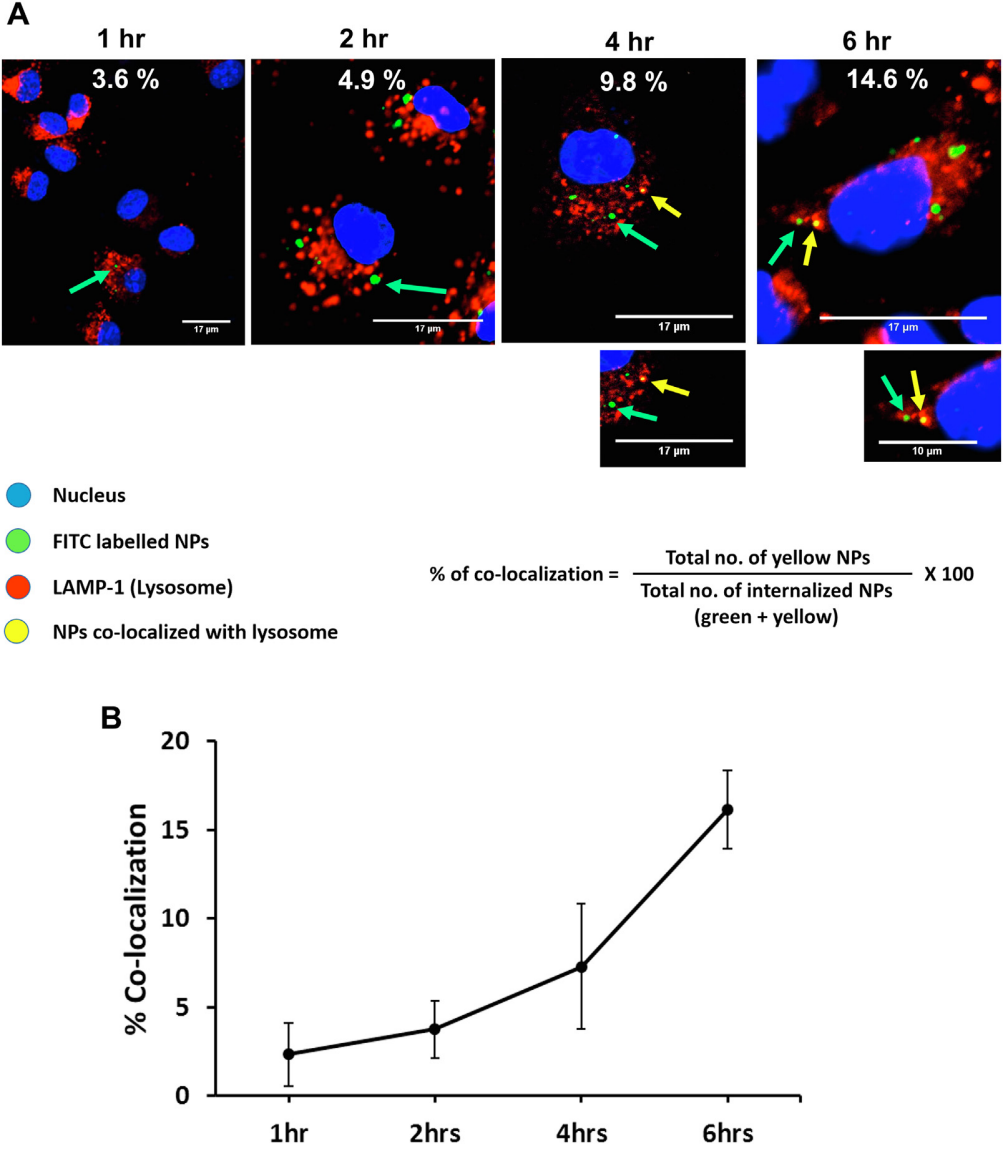
**Intracellular trafficking of E**_**91**_**NP**_***3***_**in DCs.** The DCs were cultured with E_91_NP_3_-FITC. At 1, 2, 4, and 6 h post-culture, the cells were stained with DAPI (*blue*) and fluorochrome-labeled anti-LAMP1 Abs. *A*, confocal images depict E_91_NP_3_-FITC, LAMP1, and colocalization (*arrow*) of E_91_NP_3_-FITC and lysosomes (*yellow*) at 1, 2, 4, and 6 h. The nucleus is shown as *blue*. The formula used for the calculation of the percentage of colocalization is also shown. *B*, the line graph depicts the gradual trafficking of the E_91_NP_3_-FITC to the lysosome in a time-dependent manner. Data shown at each time point are from 50 ± 5 individual cells and two independent experiments. Ab, antibody; DAPI, 4′,6-diamidino-2-phenylindole; DC, dendritic cell; LAMP1, lysosomal-associated membrane protein 1; NP, nanoparticle.

Extracellular antigens engulfed by DCs follow the exogenous pathway of antigen processing and presentation to activate CD4 T cells in context with MHC II molecules (36). The phagocytosed antigen traverses to lysosomes, where the peptide is loaded onto the MHC II molecules for display on the surface of the APCs for presentation to T cells. We incubated DCs with E_91_NP_3_-FITC and observed an augmentation in the colocalization of the lysosome (lysosomal-associated membrane protein 1 [LAMP-1]) and E_91_NP_3_-FITC through confocal microscopy in a time-dependent fashion (0–6 h) (Fig. 7, *A* and *B*). We noticed that the of colocalization increased from 3.6% (1 h) to 14.6% (6 h). This experiment suggested that there was an increase in the trafficking of E_91_NP_3_-FITC to the lysosomes in a time-dependent manner, and the acidic pH in lysosomes facilitates the release of peptides for loading onto MHC II molecules to activate specific CD4 T cells.

### Fluorescence molecular tomography tomographic live imaging of mice injected with E_91_NP_3_-FITC and priming of CD4 T cells by APCs

**Figure 8:**
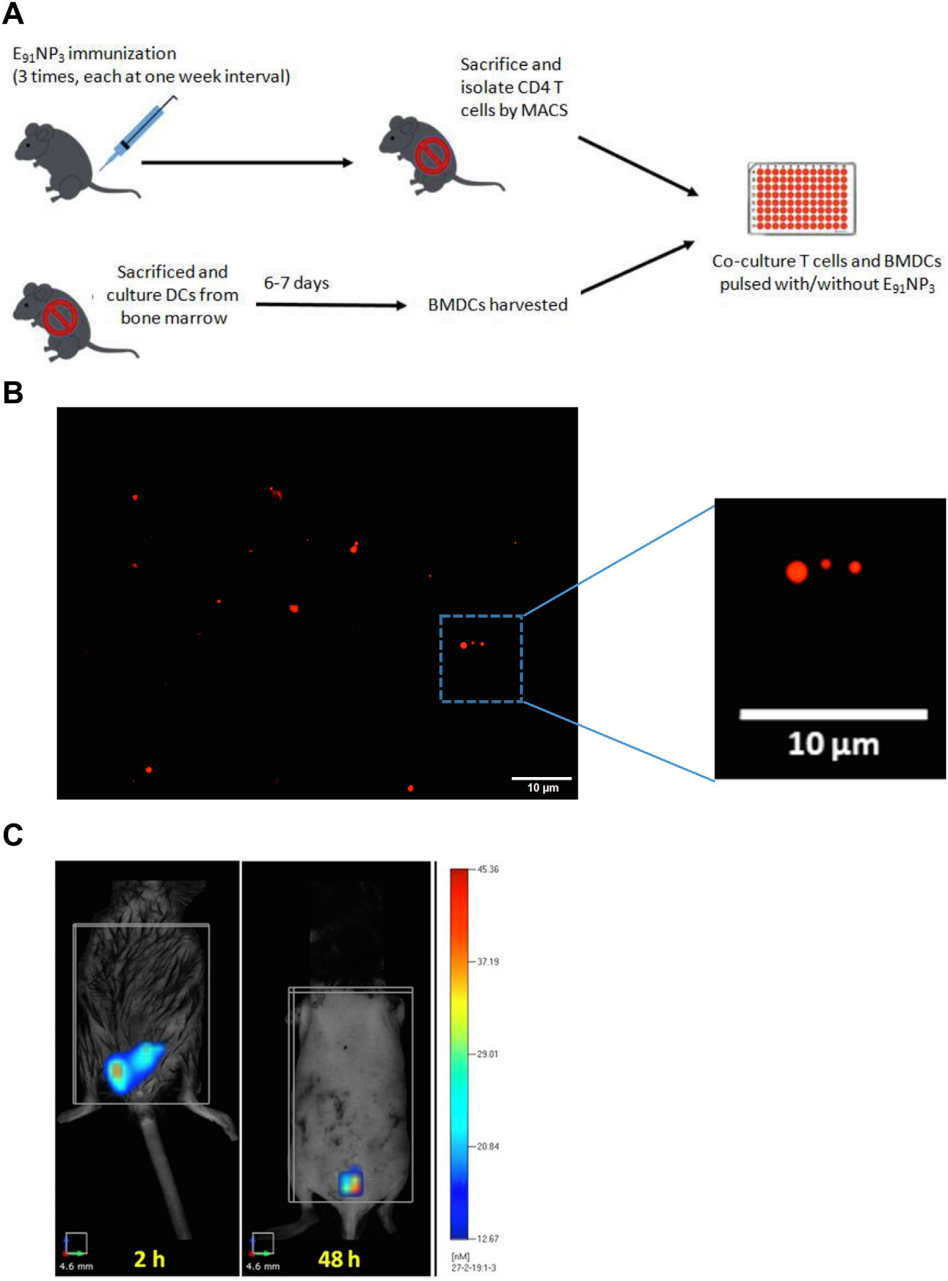

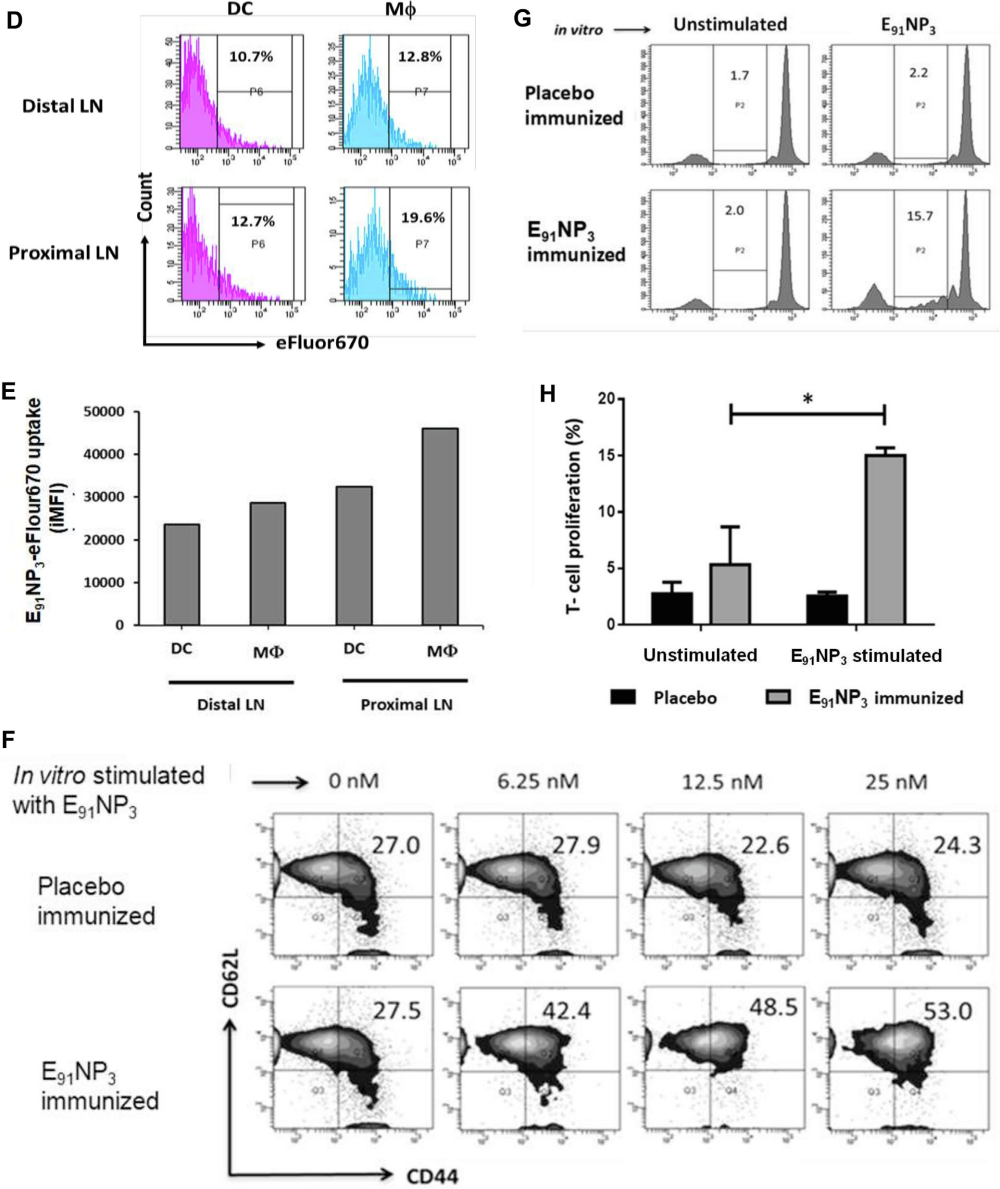

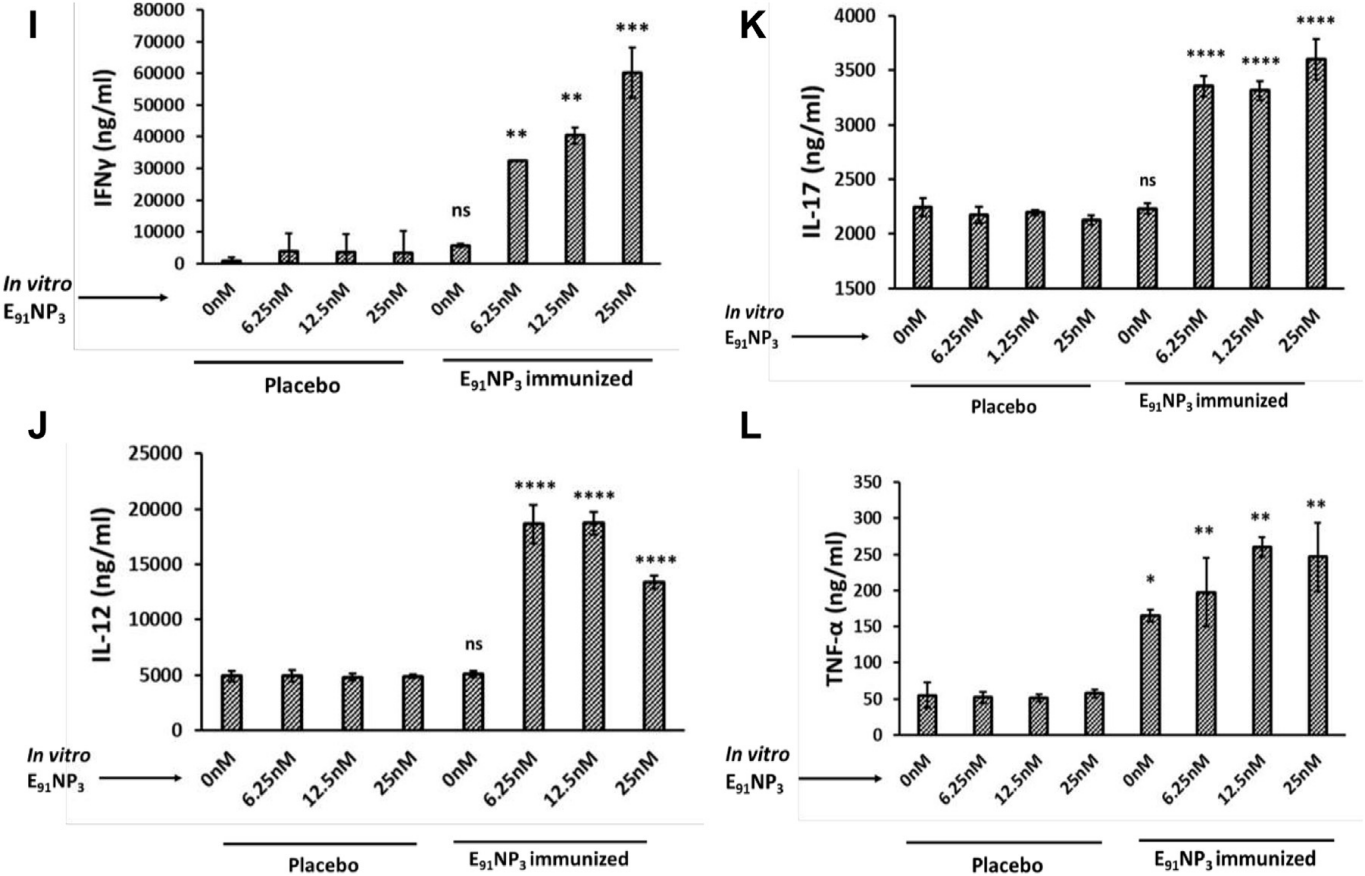
**FMT tomographic live imaging of mice injected with E**_**91**_**NP**_**3**_**-FITC and priming of CD4 T cells by APCs.** eFlour670 dye-labeled NPs (E_91_NP_3_-eFlour670) were injected s.c. in anesthetized mice. *B*, fluorescent microscopy images confirm the labeling of NPs with eFluor670. *C*, FMT images of a C57BL/6 mouse show time-dependent (2 h, 48 h, and 8 days) reduction of fluorescent intensity of E_91_NP_3_-eFlour670 from the site of injection. On day 8 postimmunization, inguinal and axillary LNs were processed, and cells isolated were stained and analyzed by flow cytometry. *D*, histograms represent a percentage. *E*, bar graph depicts the iMFI changes because of uptake of E_91_NP_3_-eFlour670 by DCs and macrophages (Mφ) to axillary (distal) LNs and inguinal proximal LNs from the site of immunization on day 8 post s.c. immunization. *A*, mice were immunized i.n. with E_91_NP_3_, followed by two boosters at intervals of 7 days. Seven days after the second booster, the animals were sacrificed. Splenocytes and LNs were pooled, and CD4 T cells were isolated. CD4 T cells were CFSE-labeled and cocultured with E_91_NP_3_ (6.25–25 nM) pulsed DCs. The cells were harvested after 72 h of incubation for flow cytometry analysis, and SNs were collected for the estimation of cytokines. *F*, contour plots represent CD4 T-cell central memory (CD44^hi^CD62L^hi^) population. *G* and *H*, the histogram and bar graph depict the proliferation of CD4 T cells stimulated with or without E_91_NP_3_. *I*–*L*, the cytokines were estimated in the SNs by ELISA. Bar diagrams represent the level (nanograms per milliliter) of (*I*) IFN-γ, (*J*) IL-12, (*K*) IL-17, and (*L*) TNF-α. The data are representative of two to three independent experiments with three mice per group. ∗*p* < 0.05, ∗∗*p* < 0.01, ∗∗∗*p* < 0.001, and ∗∗∗∗*p* < 0.0001. APC, antigen-presenting cell; CFSE, 5(6)-carboxyfluorescein diacetate *N*-succinimidyl ester; DC, dendritic cell; FMT, fluorescence molecular tomography; IFN-γ, interferon gamma; IL, interleukin; iMFI, integrated mean fluorescence intensity; LN, lymph node; NP, nanoparticle; SN, supernatant; TNF-α, tumor necrosis factor-alpha.

The mice were immunized with E_91_NP_3_ (Fig. 8*A*) or eFluor670 dye–labeled E_91_NP_3_ (E_91_NP_3_-eFlour670). The DCs migrate to the site of immunization to capture and carry the antigen to the spleen and draining LNs to prime the naïve T cells (37). Confocal imaging revealed the deposition of E_91_NP_3_-eFlour670 in the skin (Fig. 8*B*). Furthermore, we performed fluorescence molecular tomography (FMT) imaging of live animals and observed the formation of depots of E_91_NP_3_-eFlour670 at the site of injection (Fig. 8*C*). The chitosan NPs help in the slow release of the antigens (38). A gradual reduction in fluorescence intensity at the site of injection was noted. On day 8 postimmunization, flow cytometry staining and analysis of cells from inguinal (proximal) and axillary (distal) LNs showed that DCs and macrophages carried E_91_NP_3_-eFlour670 from the site of immunization to the secondary lymphoid organs. We noticed that E_91_NP_3_-eFlour670 was mostly carried to the draining (inguinal) LNs (Fig. 8, *D* and *E*). Thus, establishing the migration of the E_91_NP_3_ from the site of injection to the lymphoid organs by the APCs for the activation of antigen-specific T cells.

**Supplemental Figure 6 A,B:**
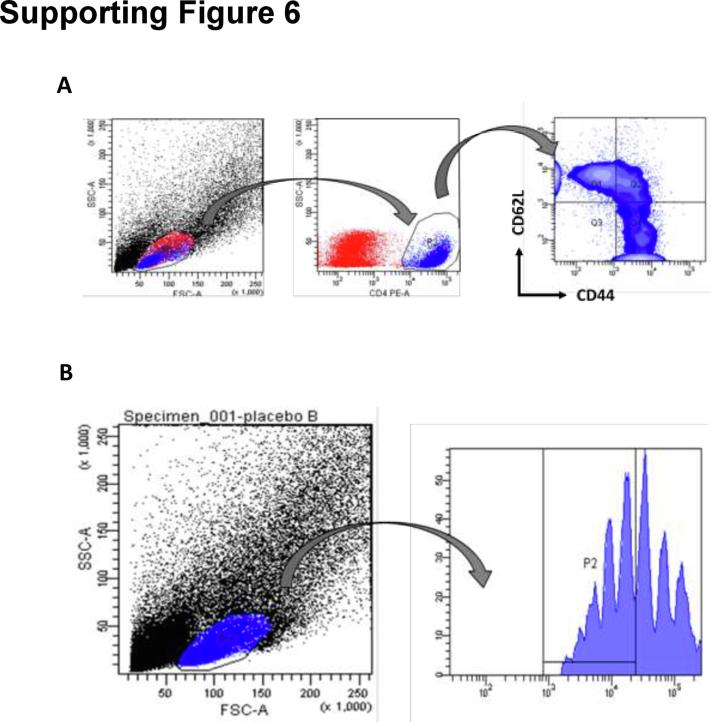


The antigens processed by DCs through the endocytic pathway are presented in context with MHC II to naïve CD4 T cells to trigger their activation, proliferation, and differentiation. Since E_91_NP_3_ is processed by the endocytic pathway, we next evaluated whether E_91_ can be associated with MHC II for the presentation to T cells. The E_91_NP_3_-pulsed DCs were cocultured with syngeneic T cells isolated from E_91_NP_3_-immunized mice (Fig. 8*A*). The gating strategy for the analysis of acquired cells in the flow cytometer is depicted in Fig. S6, *A* and *B*. We observed a dose-dependent increase in the CD4 T-cell central memory (CD44^hi^CD62L^hi^) population and their antigen-specific proliferation (Fig. 8, *F*–*H*). Furthermore, the SNs obtained from these cultures showed augmented secretion of interferon gamma (IFN-γ), IL-12, IL-17, and TNFα (Fig. 8, *I*–*L*). These results ascertained that the immunization of E_91_NP_3_ predominantly generated memory Th1 cells and Th17 cells, the cells that play a crucial role in protection against TB (7, 39, 40).

### E_91_NP_3_ activates not only CD4 T cells but also CD8 T cells

**Figure 9:**
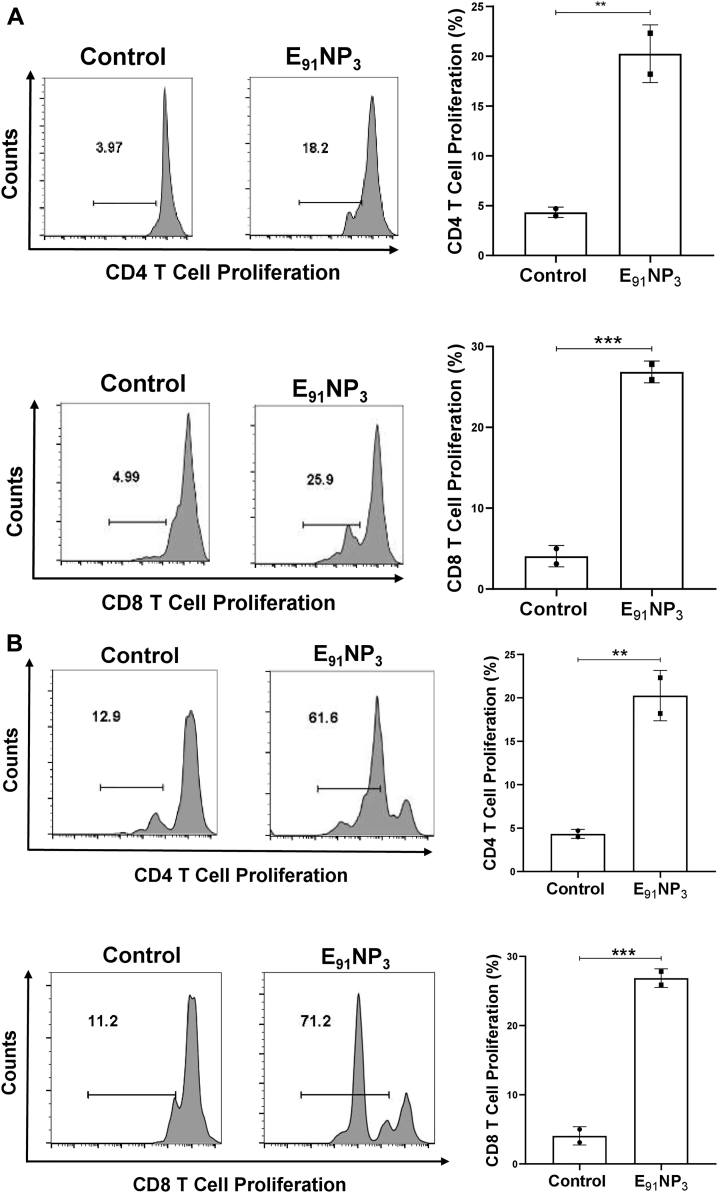

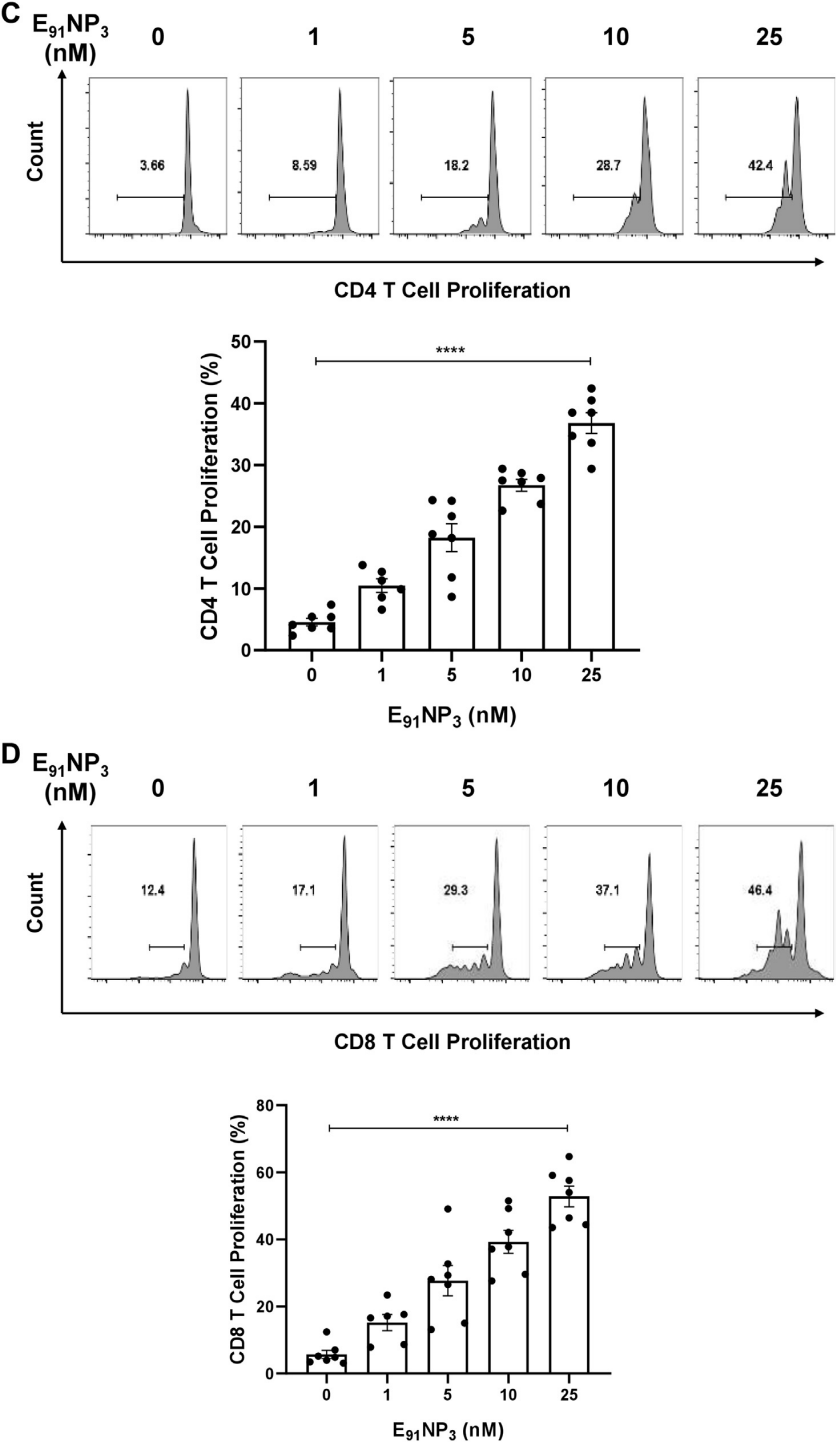
**E**_**91**_**NP**_**3**_**activates not only CD4 T cells but also CD8 T cells.** The splenocytes isolated from the mice immunized with E_91_NP_3_ were CFSE labeled. *A*, the splenocytes (1 × 10^5^/well) were cultured *in vitro* with E_91_NP_3_ (10 nM). *B*, T cells (1.5 × 10^5^/well) isolated from the splenocytes of the immunized mice were cultured with DCs (5 × 10^4^/well) and E_91_NP_3_ (10 nM). The control cultures were set without E_91_NP_3_. *C* and *D*, The PBMCs (5 × 10^4^/well) obtained from the PPD^+^ healthy subjects were cultured with E_91_NP_3_ (0–25 nM). The cells were cultured for 72 h. Later, the cells were stained with anti-CD4 and CD8 Abs and enumerated for proliferation of CD4 T cells and CD8 T cells by CFSE-dye dilution assay using flow cytometry. The data (mean ± SE) are from (*A* and *B*); each dot signifies two independent experiments (mice = three per group); (*C* and *D*) each dot indicates one volunteer (n = 7 volunteers). ∗*p* < 0.05, ∗∗*p* < 0.01, ∗∗∗*p* < 0.001, and ∗∗∗∗*p* < 0.0001. CFSE, 5(6)-carboxyfluorescein diacetate *N*-succinimidyl ester; DC, dendritic cell; NP, nanoparticle; PBMC, peripheral blood mononuclear cell; PPD^+^, purified protein derivative^+^.

The E_91_ is an HLA class II binding epitope with 20 amino acids (sequence 91–110) of the Acr1 protein of *Mtb* that activates CD4 T cells (11). We were curious to know if E_91_ can elicit CD8 T cells, as well. *In silico* analysis of E_91_ revealed that it also has MHC-I binding epitopes. Furthermore, the epitopes expressed high affinity and promiscuous binding with various HLA class I alleles (Table S1A, B). Interestingly, the lymphocytes of mice immunized with E_91_NP_3_ showed the proliferation of CD4 T cells as well as CD8 T cells (Fig. 9*A*). Furthermore, the T cells isolated from E_91_NP_3_-inoculated mice cultured with E_91_NP_3_-pulsed DCs exhibited the proliferation of both CD4 T cells and CD8 T cells (Fig. 9*B*). E_91_NP_3_ mainly elicited the production of IFN-γ (Fig. 8*I*). We observed the production of IL-10 (*p* < 0.05), but no change was noted in the levels of IL-4 and Tregs (Fig. S5, *A*–*C*). We have earlier reported that E_91_ predominantly induces the secretion of IFN-γ but not IL-4 (11).

We were curious to know whether E_91_NP_3_ would stimulate human T cells. Interestingly, E_91_NP_3_ significantly induced the proliferation of CD4 T cells and CD8 T cells obtained from the peripheral blood mononuclear cells of the BCG vaccinated and purified protein derivative^+^ (PPD^+^) donors (Fig. 9, *C* and *D*), suggesting that E_91_NP_3_ can also activate human CD4 T cells and CD8 T cells. The results suggested that E_91_NP_3_ has the potential to elicit or boost anti-*Mtb* responses in humans and therefore can be further tested in clinical trials for evaluating its efficacy.

### E_91_NP_3_ vaccination reduces the *Mtb* burden in the lungs.

**Figure 10:**
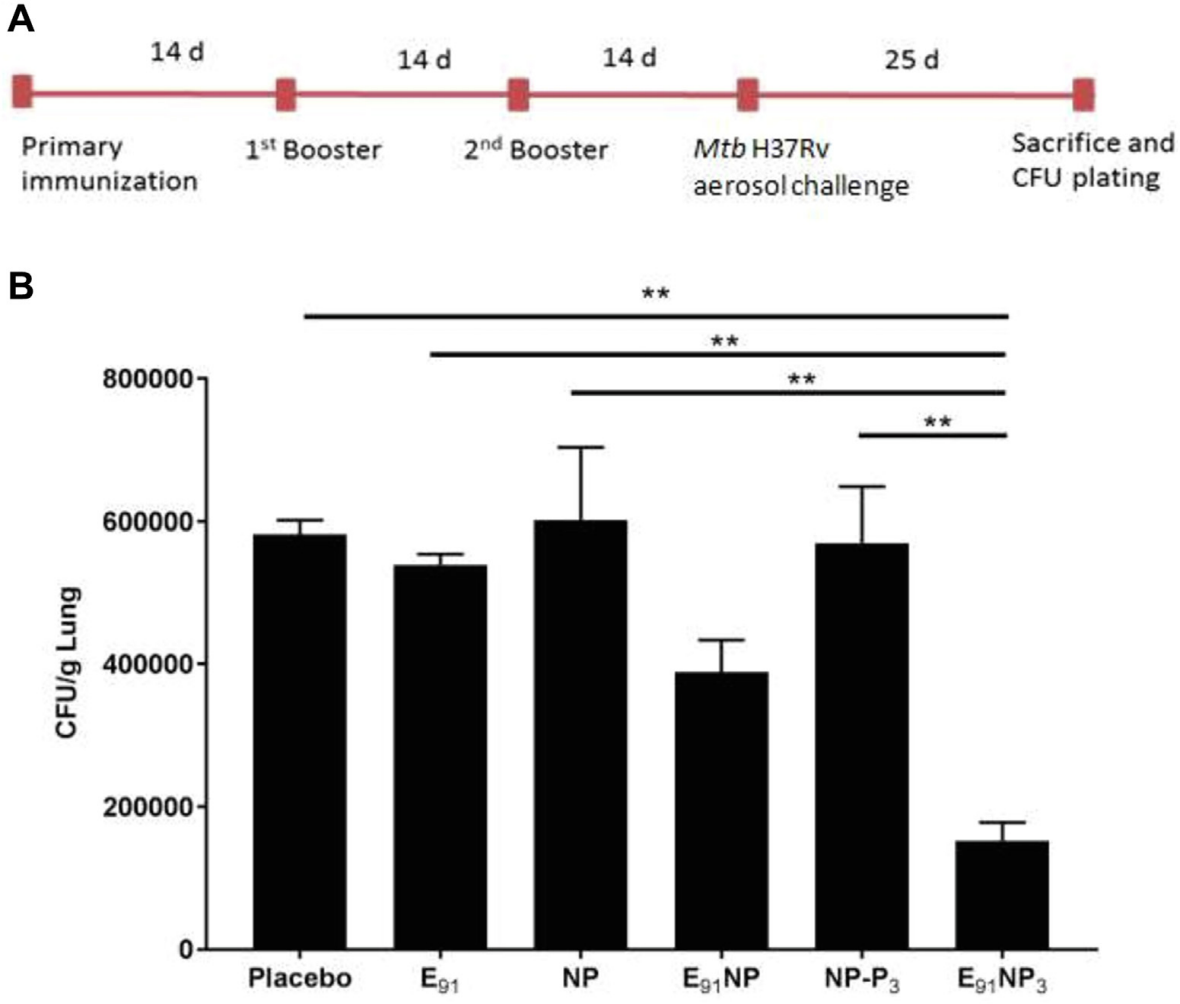
**E**_**91**_**NP**_**3**_**vaccination reduces *Mtb* burden in the lungs.** The mice were immunized (i.n.) with E_91_NP_3_. Later, two booster doses were given at an interval of 14 days. Two weeks after final immunization, the mice were aerosol-challenged with *Mtb*-H37Rv. Finally, after 25 days post-infection, the animals were sacrificed to enumerate *Mtb* burden in the lungs. *A*, schematic diagram showing the time points of immunization, infection, and experimentation. *B*, bar graph shows the colony-forming unit count/gram lung tissue for each group of mice. The data (mean ± SE) shown are representative of two experiments with three mice per group. ∗∗*p* < 0.01. *Mtb*, *Mycobacterium tuberculosis*; NP, nanoparticle.

Finally, we checked whether the E_91_NP_3_ vaccine formulation could induce a protective response in vaccinated mice in a challenge model and reduce the bacterial load. The animals previously immunized with E_91_NP_3_ were exposed to aerosolized *Mtb*, and the bacterial loads were enumerated in the lung tissues. We observed significantly reduced colony-forming units of the bacterium in the lungs, as compared with the control groups (inoculated with placebo, E_91_, NP, E_91_NP, and NP-P_3_) (Fig. 10, *A* and *B*). These results clearly showed that the immunological data induced following E_91_NP_3_ vaccination conferred protection to the host against *Mtb*.

## Discussion

Despite the fact that the BCG vaccine has been used for nearly 100 years, TB continues to infect people worldwide, which suggests that radical changes are required in the current vaccination strategies. TB is highly prevalent in TB-endemic countries. Therefore, the vaccine should not only be effective but also not require stringent storage conditions and subzero cold chains. Chitosan NPs are stable and therefore may accomplish this requirement (41). The E_91_NP_3_ were quite stable since no significant leakage of E_91_ was observed, and the average size (460 nm) was constant. These are the important parameters for the stability of the NPs (42). The NPs of sizes ranging from 200 to 1000 nm can be easily engulfed by DCs. DCs carry the NPs to the lymph nodes (LNs) to prime naïve T cells (43, 44, 45). We observed robust activation of CD4 T cells.

The mechanism of uptake deciphered of the E_91_NP_3_ by DCs was mainly through TLR-2-mediated endocytosis. Importantly, the E_91_NP_3_ after entry into the DCs were localized inside the lysosomes, where they encountered acidic pH. At low acidic pH, chitosan NPs expanded because of ionic repulsion of protonated free amino groups among the neighboring chitosan chains (46). This releases the entrapped E_91_ epitope from the NPs, leading to efficient loading of E_91_ on the MHC II molecules. E_91_NP_3_-activated DCs expressed high levels of MHC-II and costimulatory molecules and therefore exhibited better activation of T cells. The vaccine induced a specific response of CD8 T cells, Th1 cells, and Th17 cells; the cells that are crucial for imparting anti-TB immunity (39, 40, 47). Furthermore, the vaccine-elicited immune memory could be efficiently recalled to provide protection against *Mtb.* Th17 cells bolster the mucosal immunity (48, 49). E_91_NP_3_ expanded the pool of central memory CD4 T cells, and such cells could contribute to long-term protection, a critical parameter for any vaccination strategy. Furthermore, the observed proliferation of CD4 T cells and CD8 T cells of PPD^+^ subjects suggests that the E_91_NP_3_ might induce protective responses in immunized humans. We also envisage the utility of such a formulation to boost the already existing response in individuals previously exposed to *Mtb* or those immunized with BCG.

NO shows microbicidal action against *Mtb.* It suppresses NF-κB activity and promotes neutrophil recruitment through the synthesis of IL-1, IL-12, IL-15, and lipoxygenase (50, 51). Our findings revealed that E_91_NP_3_ stimulated DCs to release NO by inducing the production of iNOS, a powerful anti-*Mtb* factor. In addition, the secretion of proinflammatory cytokines, such as IL-12, IL-6, TNF-α, and IL-1, was observed. Furthermore, IL-6 boosts the effectiveness of the *Mtb* subunit vaccines (52). IL-12 plays a critical role in anti-TB immunity. Humans who lack the IL-12 gene are more vulnerable to *Mtb* infection (53). IL-12 induces DC migration to draining LNs, where T cells are primed and differentiated into effector T cells (54). IL-1β secreted by the activated DCs helps in the initial protection against *Mtb* infection (55). TNF-α is required for long-term protection and granuloma formation to control the dissemination of *Mtb* (56).

In the current study, we have used a single immunodominant epitope. Nevertheless, it is important to investigate similar NPs with peptides from different stages of infection. A combination approach of NPs encapsulating multistage epitopes of *Mtb* can also be explored to cover the latent and chronic stages of TB. Furthermore, similar NPs with wider pH stability are desired. This will help in immunizing through the subcutaneous route with larger doses, as compared with the intranasal route, possibly helping in eliciting better immune responses.

Finally, we tried to elucidate the signaling mechanism involved in eliciting the immune response by E_91_NP_3._ We noticed an enhancement in the expression of signaling molecules, MyD88, phospho-p38, and NF-kB-p65, indicating that the mechanism responsible for stimulating immunity by E_91_NP_3_ is through the TLR-2 pathway. This was further substantiated by the fact that DCs generated from TLR-2^−/−^ mice were less activated than the WT DCs with E_91_NP_3_. TLR-2 internalization into endosomes is necessary for NF-κB activation. MyD88-deficient mice are highly susceptible to *Mtb* infection (53). Furthermore, TLR-2/MyD88-dependent protection against *Mtb* is helpful in the regulation of host immunity by preventing excessive inflammation and pathogenic damage to the lungs and protecting against chronic TB infection (57). Signaling through TLR-2 amplifies IFN-γ-stimulated NO release in conjunction between MyD88 and IFN-γR (18).

Our results illustrate that E_91_NP_3_ evokes immunity essential to protect against *Mtb*. This was further confirmed when it was noted that animals vaccinated with E_91_NP_3_ demonstrated a remarkable reduction in the *Mtb* burden in their lungs. Overall, these results suggest that in the future, E_91_NP_3_ may be a potential vaccine candidate against TB.

## Experimental procedures

### Chemicals and reagents

Chitosan (catalog no.: 448869), pentasodium tripolyphosphate hexahydrate (TPP) (catalog no.: T5633), *N*-hydroxylsuccinimide (NHS) (catalog no.: 130672), *N*-(3-dimethylaminopropyl)-*N*′-ethylcarbodiimide hydrochloride (EDC) (catalog no.: E-1769), collodion solution (catalog no.: 09986), PEG3350 (catalog no.: 202444), and PEG1000 (catalog no.: 81188) were purchased from Sigma–Aldrich. E_91_ peptide of >95% purity (sequence: SEFAYGSFVRTVSLPVGADE) was synthesized by GLBiochem. Pam3CSK4 (tlrl-pms) and biotinylated P_3_ (tlrl-bpms) were procured from InvivoGen. MES buffer (catalog no.: RM1128) and trehalose (catalog no.: GRM110) were purchased from HiMedia. Sucrose (catalog no.: 84973), d-mannitol (catalog no.: 79887), and d-glucose (catalog no.: 42738) were from SRL. Propidium iodide (PI; catalog no.: P4170), paraformaldehyde (catalog no.: P6148), dynasore hydrate (catalog no.: T9424), chloroquine diphosphate salt (catalog no.: C6628), chlorpromazine hydrochloride (catalog no.: C8138), genistein (catalog no.: G6649), nystatin (catalog no.: N6261), cytochalasin D (catalog no.: C8273), rottlerin (catalog no.: R5648), 5(6)-carboxyfluorescein diacetate *N*-succinimidyl ester (CFSE) (catalog no.: 21888F), and incomplete Freund’s adjuvant (catalog no.: F5506) were purchased from Sigma–Aldrich. Abs for flow cytometry were as follows: CD16/CD32 (BD Biosciences; catalog no.: 32553142), CD11c PECy7 (BD Biosciences; catalog no.: 117318), MHC II PerCpCy5.5 (BioLegend; catalog no.: 116416), CD86 PE (eBiosciences; catalog no.: 12-0862-85), CD40 APC (BD Biosciences; catalog no.: 558695), CD4 PE (BioLegend; catalog no.: 100512), and CD44 PerCPCy5.5 (eBiosciences; catalog no.: 45-0441-82). All Abs for ELISA, granulocyte–macrophage colony-stimulating factor (GM-CSF; catalog no.: 550068) and IL-4 (catalog no.: 550067) were procured from BD Pharmingen. Abs used in Western blotting were iNOS (Abcam; catalog no.: ab3523), p65 NF-κB (Cell Signaling Technology; catalog no.: 8242), MyD88 (Cell Signaling Technology; catalog no.: 4283), p38 (Cell Signaling Technology; catalog no.: 9212), phospho-STAT3 (Cell Signaling Technology; catalog no.: 9131), STAT3 (Cell Signaling Technology; catalog no.: 30835), and β-actin (Sigma–Aldrich; catalog no.: A1978). Other chemicals and solvents used were commercially available and of a high degree of purity. RPMI-1640 media were purchased from Invitrogen, Life Technologies, fetal calf serum (FCS) from Gibco, l-pyruvate (SERVA; catalog no.:15220), l-glutamine (SERVA; catalog no.: 22942), streptomycin (SERVA; catalog no.: 35500), and penicillin (SERVA; catalog no.: 31749). The primers for RT–qPCR were purchased from Sigma–Aldrich. All tissue culture–grade plastic wares were procured from BD Biosciences, Thermo Fisher Scientific, Corning, and Sigma–Aldrich. Other chemicals and solvents used were commercially available and of a high degree of purity.

### Synthesis of blank NPs and E_91_-entrapped NPs

NPs were prepared by the ionic gelation method with TPP as described elsewhere with slight modifications (58). Briefly, 10 mg chitosan powder was dissolved in a solution containing 10 ml of distilled water and 100 μl of glacial acetic acid, and the mixture was kept under constant stirring for 2 h. The pH of the chitosan solution was adjusted to 5.0 using a solution of 10 N NaOH. Meanwhile, TPP solution of 1 mg/ml concentration was prepared, and pH was adjusted to 2.0 using 1:10 HCl solution. The TPP solution (1 mg/ml) was added dropwise to the chitosan solution under stirring. The solution is kept under stirring for another 4 to 5 h. The prepared chitosan NPs were dialyzed overnight against the MES buffer (pH 5, 10 mM) and used for various characterizations. The NPs can be harvested by centrifuging at 14,000*g* for 30 min at 10 °C.

### Analysis of NP size distribution by DLS and ζ-potential

The size distribution of the synthesized carbon NPs was evaluated by DLS using a Malvern Ζ-sizer Nano ZS instrument (Malvern Instruments). The readings were performed in automode for up to 100 scans in three independent readings in disposable polystyrene cuvettes at 25 °C, with measurement preceded by 30 s equilibration time. The surface ζ-potential of the NPs was measured in the same instrument using a folded capillary ζ-cell. ζ-potential denotes the potential of the NPs at the surface of the Stern layer, and a value <−30 mV or >+30 mV denotes a stable colloidal suspension. The readings were performed in automode for up to 100 scans in three independent measurements at 25 °C, preceded by 60 s equilibration time.

### Analysis of NP morphology and estimation of size by FE-SEM and TEM

The morphology and size of the synthesized NPs were analyzed using TEM (JEM-2100CR). Sample preparation was done 1 h prior to acquiring. Shortly, NP suspension was drop coated over carbon-coated copper grids (300 mesh) (Polysciences) and kept under a light bulb at room temperature (22 ± 2 °C) within a petri plate. For FE-SEM, the NP sample was drop cast on a clean glass slide under a laminar hood. The sample was kept overnight at 37 °C in a dry environment. The dried samples were used for acquiring in the Hitachi-SU8000 FE-SEM instrument under slow scan speed and using an accelerating voltage of 5000 V and a decelerating voltage of 0 V.

### Analysis of NP morphology and estimation of size by AFM

The morphology, size, and surface topology of the synthesized NPs were analyzed using AFM (Veeco; model: XE100). Prior to the deposition of samples, the mica sheet was freshly peeled using cellophane tape under a laminar hood. The NP solution was carefully mounted by drop casting on the freshly cleaved mica sheet. The mica sheet was covered inside a petri dish and kept at 4 °C overnight. Prior to sample acquisition, the mica sheet was slowly washed with deionized water (18 MΩ) under a slanting position and dried under a slow jet of argon gas at 22 ± 2 °C. The prepared sample was then mounted onto the instrument for imaging. The images were acquired in the tapping mode using silicon nitride tips (Tap300AI-G; Budget sensors) at a resonance frequency of 300 kHz and a spring constant of 40 N/min. The scanning rate was 9.8446 μm/s. All images were processed using SPIP software.

### Stability studies of NPs

The NPs were incubated at 4 °C or at room temperature for up to 5 months, and the hydrodynamic size of the NPs was measured by using the DLS instrument at 2, 6, 15, 30, 60, 90, 120, and 150 days. For storage at −80 °C, the NPs were incubated with different cryoprotectants, namely sucrose, d-glucose, trehalose, mannitol, PEG1000, and PEG 3350 (0, 6.25, 12.5, 25, and 50 μg/ml). The frozen samples were later thawed, and hydrodynamic sizes were measured again.

### Preparation of E_91_ encapsulated NPs (E_91_NP)

The following E_91_:chitosan ratios were used for entrapment studies: 40 μg: 1 mg, 80 μg: 1 mg, and 160 μg: 1 mg. The corresponding sizes and ζ-potential changes because of different peptide loadings were also measured. To calculate the EE, 1 ml of the E_91_NP solution was centrifuged at 14,000*g* for 30 min. The SNs were used for the estimation of free E_91_ peptide by BCA assay. Free E_91_ was used for plotting standard curves. The following formula was used to calculate the EE:

$$EE=\frac{\left(initial\;E91-free\;E91\;in\;supernatant\right)}{initial\;E91}\times 100\%$$

### Thermogravimetric analysis

Thermogravimetric analysis (TGA) was performed using TGA/DSC (Mettler Toledo; model: TGA/DSC-l). About 5 mg of powdered samples were mounted on the TGA/DSC sample pan having 1 μg resolution without range switching. The run was performed at a temperature range of 25 to 800 °C within a 90 min time frame. DTA is plotted using the STARe software (Mettler Toledo).

### Release kinetic studies of NPs

For studying the release kinetics profile of the E_91_ peptide, the particles were dialyzed against the MES buffer (pH 5, 10 mM) and kept for 20 days. One milliliter of the NP suspension was taken out at regular intervals and replaced with a fresh buffer. The collected samples were centrifuged at 14,000*g* for 30 min. The SNs were collected at each time point, centrifuged, and BCA estimation was performed on the SNs. Free E_91_ was used for plotting standard curves.

### Conjugation of P_3_ on NP surface

EDC/NHS coupling chemistry was used to link 1° amine with carboxyl to yield amide bonds. Chitosan polymers rich in 1° amine were utilized to link it with the carboxyl group of P_3_. This carboxyl group does not take part in binding to TLR-1/TLR-2; hence, it can be utilized for conjugation. The P_3_ molecule is first activated with EDC/NHS 2:1, and then, the NPs in MES buffer (pH 6, 100 mM) are used in the conjugation procedure.

### Stability of E_91_NP_3_

The E_91_NP_3_ were kept for 30 days at 4 °C. Later, the SNs were collected, and leakage of E_91_ peptide was quantified by the Bradford method. Furthermore, the entrapment of E_91_ in E_91_NP_3_ was demonstrated by disrupting E_91_NP_3_ by ultrasonication, and the presence of E_91_ was estimated. The E_91_NP_3_ were stable for 30 days.

### ITC to confirm the conjugation of P_3_ on the NP surface

ITC was performed to confirm the conjugation of P_3_ on the NP surface. For this, commercially available biotinylated P_3_ (bioP_3_) was used for the conjugation reaction in place of P_3_. However, unbiotinylated P_3_ was kept as a control along with all buffer controls. Once the bioP_3_ or P_3_ was conjugated to the surface of the NPs, the conjugate was dialyzed to remove any unbound bioP_3_ or P_3_. Prior to the experiment, NP-bioP_3_ or NP-P_3_ as well as HRP–streptavidin were dialyzed against MES buffer (10 mM, pH 5.5). Degassing was performed by centrifugation at 21,000*g* in Fresco 21 centrifuge (Thermo Fisher Scientific) before the experiments. The ITC experiment was performed at 25 °C using MicroCal VP-ITC (GE Healthcare Bio-Sciences AB). The injection volume and reference power were kept at 5 μl and 6 μcal·s^−1^, respectively, and a constant stirring speed of 286 rpm was maintained in the sample cell throughout the experiment. Control experiments were performed under the same experimental conditions. The one-site model was used to analyze protein–ligand binding. The data were analyzed using the Origin 6.0 software suite (Origin Lab).

### Modified ELISA to confirm the conjugation of P_3_ on NP surface

Cellulose nitrate was coated on 96-well plate surfaces by incubating the plate at 37 °C for 1 h. Cellulose nitrate provides a negative surface on the microplate surface. The positively charged NP/biotin-P_3_ were coated on the negative surface at log_2_ dilutions at 4 °C overnight. Thereafter, avidin–HRP was poured into the wells and incubated at 37 °C. Proper washings were done with a phosphate buffer in between these steps to avoid nonspecificity. The development of color was performed using o-Phenylenediamine as a chromogen substrate. The reaction was stopped with 7% H_2_SO_4_, and the absorbance was measured at 492 nm.

### FTIR

Lyophilized NP and NP-P_3_ samples were finely grounded with FTIR-grade potassium bromide and dehydrated in the presence of CaCl_2_ inside a vacuum desiccator, and pellets were prepared just before acquiring by using a hydraulic die casting machine. The samples were acquired in FTIR Spectrophotometer (Bruker Vertex-70) with a scanning range of 350 to 4000 cm^−1^ and a scan speed of 15 spectra/s at cm^−1^.

### Ethical statement

Female WT C57BL/6 and TLR-2^−/−^ mice (aged 6–8 weeks) were procured from the Animal House of the Institute of Microbial Technology (IMTECH), Chandigarh and Indian Institute of Science Education and Research (IISER), Mohali, India. The study was approved by the Institutional Animal Ethics Committee of IMTECH (no.: 55/1999/CPCSEA) and IISER (no.: 1842/GO/ReBibt/S/15/CPCSEA), Ministry of Environment and Forest, Government of India. The BCG-vaccinated and PPD^+^ healthy volunteers (age: 23–60 years) were involved in the study. The experiments were approved by the Institutional Ethics Committee of the Indian Institute of Technology, Ropar. The experiments were done in accordance with the ethical guidelines for biomedical research on human subjects by the Central Ethics Committee on Human Research (CECHR), ICMR-2000, and those as contained in “The Declaration of Helsinki.” The donors gave in writing informed consent.

### Mycobacterial strain

*Mtb* H37Rv strain was a kind gift from the National JALMA Institute of Leprosy and Other Mycobacterial Diseases, Agra, India. *Mtb* H37Rv was cultured in a 7H9 medium containing Tween-80 (0.05%) supplemented with oleic acid, albumin, dextrose, and catalase. Glycerol stocks of H37Rv were prepared and stored at −80 °C for infection studies.

### DC cultures

The methodology for culturing bone marrow–derived DCs was followed according to the protocol mentioned by Lutz *et al.* (59). Briefly, bone marrow cells were isolated from the femurs and tibiae of WT and TLR2^−/−^ mice. The bone marrow cells were flushed aseptically from the femurs and tibia. The cells were counted, and 2 × 10^6^ cells per well were plated onto 6-well plates. For DC cultures, cells were grown in RPMI-1640 containing FCS (10%) supplemented with penicillin (100 U/ml), l-glutamine (100 mM), streptomycin (100 mg/ml), and GM-CSF(2 ng/ml), and rIL-4 (4 ng/ml). On the third day, the culture was replenished with 50% of the initial volume with fresh media containing GM-CSF (2 ng/ml) and rIL-4 (4 ng/ml). These cells were maintained for another 3 days in a humidified atmosphere, CO_2_ (5%) at 37 °C. Loosely adherent cells were harvested by gentle pipetting on sixth/seventh day.

### NO assay

Culture SNs were collected after 24 h of incubation of DCs with E_91_NP_3_ and controls (DCs + NPs, DCs + E_91_, DCs + P_3_, and DCs). NO was measured by the Griess method (60, 61). Briefly, SNs (50 μl) were incubated with an equal volume of Griess reagent for 5 min at room temperature (22 ± 2 °C). Later, the absorbance was measured at 540 nm.

### PI assay

DCs cultured with E_91_NP_3_ (7.8–4000 nM) controls (DCs + NPs, DCs + E_91_, DCs + P_3_, and DCs) for 24 h were harvested. Thereafter, cells were incubated with 2 μl of PI (50 mg/ml) for 10 min at room temperature (62). The cells were immediately assessed through flow cytometry using FACS Accuri (BD Biosciences).

### 3-(4,5-Dimethylthiazol-2-yl)-2,5-diphenyltetrazolium bromide tetrazolium assay

3-(4,5-Dimethylthiazol-2-yl)-2,5-diphenyltetrazolium bromide tetrazolium assay was performed as reported elsewhere with slight modifications (63). Briefly, human embryonic kidney-293 cells (1 × 10^4^ cells, 96-well plate) were seeded along with E_91_NP_3_ (62.5–8000 nM) and controls (DCs + NPs, DCs + E_91_, DCs + P_3_, and DCs) for 24 h. Ethanol-fixed cells were taken as positive controls. Thereafter, the cells were treated with (3-(4,5-dimethylthiazol-2-yl)-2,5-diphenyltetrazolium bromide) tetrazolium solution to attain a final concentration of 0.5 mg/ml. The plates were incubated for 4 h in a humidified atmosphere (37 °C, 5% CO_2_). Finally, 100 μl of the solubilization solution was added per well to dissolve the formazan crystals. The plates were read at 570 nm using Synergy H1 Hybrid Multi-Mode Reader (BioTek, Agilent Technologies).

### Quantification of cytokine secretion by ELISA

The secretion of cytokines in the culture SNs was estimated by sandwich ELISA as per the manufacturer’s instructions (BD Pharmingen). Briefly, the ELISA plates were coated with Abs against IFN-γ, IL-6, IL-12, IL-4, and IL-10 overnight at 4 °C. The next day, the plates were washed to remove unbound Abs. Furthermore, unbound sites were blocked with 1% bovine serum albumin (BSA). Subsequently, culture SNs were added along with the respective standards for the cytokines. Following overnight incubation, the samples were treated with biotinylated capture Abs against their respective cytokines for 2 h. Later, following proper washing for removing the unbound Abs, incubation with HRP–streptavidin was done for 1 h at room temperature. The development of color was performed using o-phenylenediamine as a chromogen substrate. The reaction was stopped using 7% H_2_SO_4_. The absorbance was measured at 492 nm. Usual steps of washing and incubation were performed at each step using PBS with Tween-20 buffer. The quantification of secreted cytokines in the culture SNs was done using the standard plot generated from the recombinant cytokines of known concentration and expressed as picogram per milliliter.

### Demonstration of the phenotype of DCs by flow cytometry

DCs (5 × 10^5^) were collected in tubes followed by washing with 1× PBS supplemented with 2% fetal bovine serum. The cells were then treated with Fc receptor blocking Abs, followed by staining with fluorochrome-conjugated anti-CD11c, CD40, CD86, and MHC II Abs, as per the recommended protocol. The cells were acquired in BD-FACSVerse, and analysis was performed using DIVA software (BD Co).

### The mechanism of NP uptake by DCs

The FITC-conjugated E_91_ was encapsulated in NPs coated with P_3_ (E_91_NP_3_-FITC). The DCs were pretreated with dynasore hydrate (10–80 μM), genistein (25–200 μM), and cytochalasin D (0.625–5 μM) and incubated for 3 h. These inhibitors are corresponding to CME (inhibitor of CME), CavME (inhibitor of CavME), and phagocytosis, respectively. The concentrations used in the experiments were according to the manufacturer's instructions. Thereafter, cells were incubated with E_91_NP_3_-FITC for 24 h. Later, cells were harvested and thoroughly washed with PBS for complete removal of any residual extracellular NPs. The DCs were first treated with Fc receptor blocking Abs, followed by staining with fluorochrome-conjugated anti-CD11c, as per the recommended protocol. The usual steps of incubation and washing were followed at each step. The cells were acquired using BD FACS Verse, and analysis was performed using BD DIVA software (San Jose, CA).

### The uptake of E_91_NP_3_-FITC by DC^TLR2−/−^

The DCs (2 × 10^6^/well) generated from WT (DC^WT^) and TLR-2^−/−^ (DC^TLR-2−/−^) C57BL/6 mice were cultured with FITC-labeled-E_91_NP_3_ (10 nM) in 6-well plate for 4 h. Later, the cells were harvested and monitored for the uptake of FITC-labeled-E_91_NP_3_. The cells were also stained for the expression of MHC-II, CD80, CD86, and CD40 using fluorochrome-tagged respective Abs. The cells were acquired using BD Accuri, and analysis was performed using BD FlowJo software (San Jose, CA).

### E_91_NP_3_-FITC uptake by DCs through confocal microscopy

The DCs were harvested on day 7 and seeded on sterile coverslips in 6-well plates (2 × 10^6^ cells/well, RPMI-1640, 10% FCS). The cells were incubated for 4 h/5% CO_2_. Thereafter, E_91_NP_3_-FITC were added to the cultures. The cells were harvested periodically at 1, 2, 4, and 6 h and washed (2×) with PBS to remove any extracellular E_91_NP_3_-FITC. The cells were fixed in 4% paraformaldehyde for 15 min and washed (3×) with PBS. The fixed cells were treated with 0.1% Triton X-100 for 90 to 120 s and immediately washed (3×) with 1× PBS. Next, the wells were blocked in 3% BSA (prepared in 1× PBS) for 1 h at 22 ± 2 °C followed by washing (2×) with 1× PBS. Later, for each time point, the wells were incubated with α-LAMP1 Abs as per the manufacturer's instructions. Thereafter, the plates were incubated at room temperature for 1 h and then washed (5×) with 1× PBS. Subsequently, cells were treated with Alexa Fluor 561–tagged anti-rabbit Ab and incubated for an additional 1 h. The wells were washed (3×) for complete removal of any unbound Abs. The cells were incubated with 4′,6-diamidino-2-phenylindole (1 μg/ml) for 10 min for staining the nucleus. The wells were then thoroughly washed (3×) with 1× PBS and mounted on glass slides using a mounting solution. The cells were monitored for antigen uptake using Nikon A1 confocal laser microscope (Nikon). The analysis was performed using National Institutes of Health ImageJ software.

### Isolation of lymphocytes from spleen, LNs, and lungs

Spleens and LNs obtained from immunized mice were pooled. A single-cell suspension was prepared by gently pressing through the frosted slides. The lungs were perfused with chilled PBS–heparin solution, and small pieces of them were prepared and digested with collagenase (2 mg/ml) for 30 min/37 °C. Later, cells were passed through a sieve (70 ml). The viability of the cells was enumerated by the trypan blue exclusion method. The cells (2 × 10^5^/well) were added to 96-well U-bottom plates and cultured with E_91_NP_3_ (6.25–25 nM).

### T-cell proliferation assays

The cells (2 × 10^5^/well) were incubated with CFSE dye (2 μM) in 1× PBS (4 ml) at 37 °C. Free CFSE was quenched with 2 ml of FCS, and excess dye was removed by washing with RPMI–FCS (10%). CFSE-labeled cells were cultured with/without E_91_NP_3_ (6.25–25 nM), and the plates were incubated for 72 to 96 h (37 °C/5% CO_2_). The SNs were collected and stored at −80 °C for ELISA. The cells were harvested and stained with fluorochrome-labeled anti-CD4 and CD8 Abs for flow cytometry using BD Accuri flow cytometer and analyzed on BD FlowJo software.

### The prediction of CD8 T-cell epitope by *in silico* method in CD4 T-cell E_91_ epitope (sequence 91–110) of Acr1 antigen of *Mtb*

We used the immune epitope database (IEDB) MHC class I immunogenicity prediction tool for identifying CD8 T-cell epitopes in the E_91_ peptide of the Acr1 antigen of *Mtb*. The tool provides a score, which indicates the probability of generating a CD8 T-cell immune response. The query sequence was entered in FASTA format in the provided interface. Using the default parameters, the sequence was submitted for prediction. The steps were repeated for each query peptide, and the results were recorded. A higher score indicates more potential to elucidate an immune response. The tool can be accessed from URL: http://tools.iedb.org/immunogenicity/.

#### CD8 T-cell epitope prediction

NetMHCpan-4.1 server was used to determine the CD8 T-cell epitopes in E_91_ peptide. The server was used for the prediction of peptide binding to the MHC/HLA class I alleles. The whole E_91_ peptide and overlapping nine mer sequences were used in FASTA format. Default parameters along with nine mer peptide length and HLA class I supertype representative were used, and the data were recorded for each peptide. The threshold for strong and weak binders was set to 0.5% and 2% rank, respectively. Binding affinity represents the attachment of peptide to the MHC I allele. The tool can be accessed from https://services.healthtech.dtu.dk/service.php?NetMHCpan-4.1.

### *In vivo* generation of Tregs

The splenocytes isolated from the E_91_NP_3_-immunized mice were stimulated *in vitro* with E_91_NP_3_ (5 nM) for 72 h. Later, the SNs were collected for the estimation of IL-4 and IL-10 by the ELISA. The cells were stained with fluorochrome-conjugated anti-CD4 Abs followed by intracellular staining with fluorochrome-conjugated anti-Foxp3, IL-10, and IL-4 Abs (Biosciences). The regular steps of incubation and washings were followed, and the cells were acquired using BD Accuri and analyzed by BD FlowJo software.

### The proliferation of human CD4 T cells and CD8 T cells

The peripheral blood mononuclear cells were isolated by the density gradient method from the 7.5 ml blood obtained from BCG-vaccinated PPD^+^ healthy volunteers in a sterile vacutainer. The blood was layered on 2.5 ml HiSep (HiMedia—HiSep LSMTM 1077-LS001) in 15 ml tube and centrifuged at 400*g* for 30 min at 25 °C. The mononuclear cells were isolated, washed, CFSE-labeled, and cultured with E_91_NP_3_ (0–25 nM) for 72 h in RPMI–FCS (10%) at 37 °C/5% CO_2_. During culture, IL-2 (100 U/ml) was added after 24 h. Later, cells were harvested and stained with fluorochrome-tagged antihuman CD4 and CD8 Abs, and proliferation was measured using flow cytometer.

### Monitoring the signaling pathway of DCs incubated with E_91_NP_3_ by Western blotting

DCs (2.5 × 10^6^ cells/well, 6-well plate) were treated with E_91_NP_3_ for 3 to 6 h. Free P_3_ treatment was taken as a positive control. The cells were harvested, washed, and lysed in a cytosolic extraction lysis buffer (with PMSF and protease and phosphatase inhibitor cocktail). The cytosolic proteins were then quantified and subjected to SDS-PAGE. After transfer onto polyvinylidene difluoride membrane; the blocking of unsaturated sites was done with 2% BSA. The membrane was then immunoblotted with Abs against iNOS, p65 NF-κB, MyD88, phospho p38 and p38, phospho STAT3 and STAT3. β-actin was used as a loading control. Regular incubation and washing were performed at each step. Blots were developed using a chemiluminescence kit (Bio-Rad). Scanning of the blots was completed with ImageQuant LAS 4000 (GE Healthcare). The image analysis was achieved with ImageJ analysis software.

### RT–qPCR for the quantification of IL-6, IL-12, TNFα, and IL-1β

Total RNA was isolated from DCs stimulated with E_91_NP_3_ and controls for 6 h by Trizol reagent according to the manufacturer’s instructions (Invitrogen). RNA was quantified with the help of a NanoDrop spectrophotometer. Absorbance at 260 nm/absorbance at 280 nm ratio of all samples was in the range of 1.90 to 2.00. DNA contamination from RNA samples was removed by amplification-grade DNase. Briefly, RNA samples (1 μg) were incubated with DNase (1 U) for 15 min in the reaction buffer. After incubation, DNase activity was terminated by a stop solution. Furthermore, the samples were heated to 70 °C for 10 min to inactivate DNase activity. Results are represented in the form of relative expressions. Analysis was performed by comparative Ct method, whereas Ct values were normalized against housekeeping control β-actin. Using the comparative Ct method, relative gene expression was calculated as 2(−ΔΔCt), where ΔCt = Ct (gene of interest) - Ct (normalizer = β-actin) and the ΔΔCt = ΔCt (sample) - ΔCt (calibrator). RT–qPCR and data analysis were done by ABI 7500 Fast Real-time PCR system (Applied Biosystems, Chromos).

*β-actin*: Forward: 5′-AGAGGGAAATCGTGCGTGAC-3′

Reverse: 5′-CAATAGTGATGACCTGGCCGT-3′

*Il-6*: Forward: 5′-GAGGATACCACTCCCAACAGACC-3′

Reverse: 5′-AAGTGCATCATCATCGTTGTTCATACA-3′

*Il-12*: Forward: 5′-GGAAGCACGGCAGCAGCAGAATA-3′

Reverse: 5′-AACTTGAGGGAGAAGTAGGAATGG-3′

*Il-1β*: Forward: 5′-CAACCAACAAGTGATATTCTCCATG-3′

Reverse: 5′-GATCCACACTCTCCAGCTGCA-3′

### DC-T cell coculture assay

The immunization scheme for this experiment is shown in Figure 8*A*. Briefly, E_91_NP_3_ (containing 10 nM E_91_) was immunized s.c. in C57BL/6 mice (three mice/group). Two boosters with E_91_NP_3_ were given intervals of 1 week. The DC culture was preset 6 days before the experiment. Seven days after the third immunization, the mice were sacrificed; splenocytes and LNs were processed. The CD4 T cells were purified by magnetic beads cell sorter, as per the manufacturer’s instructions. Briefly, after preparing single-cell suspension, the cells were treated with a CD4 T-cell enrichment cocktail. Later, the same volume (5 μl per 1 × 10^6^ cells) of streptavidin magnetic beads was added, and cells were isolated using BD IMagnet. Purified CD4 T cells were cocultured with DCs (2 × 10^5^/well, 96-well plate) (DC:T cell, 1:5). The DC–T cells were stimulated with or without E_91_NP_3_ (6.25–25 nM), and the cultures were incubated for 72 to 96 h (37 °C, 5% CO_2_).

T cells (1.5 × 10^5^/well) were isolated using magnetic beads cell sorter from the splenocytes + LNs of the E_91_NP_3_-immunized mice. These T cells were CFSE labeled and cultured with E_91_NP_3_ (10 nM)-pulsed DCs (5 × 10^4^/well) in a ratio of 5:1 for 72 h in 96-well plate. The control cultures were set without E_91_NP_3_. Later, the cells were harvested and stained with anti-CD4 and CD8 Abs and enumerated for proliferation by flow cytometer.

### *In vivo* FMT tomographic imaging of animals

For *in vivo* imaging, eFluor670 dye–labeled NPs were injected s.c. in C57BL/6 mice. NPs labeling with eFluor670 were confirmed by fluorescent microscopy. Prior to *in vivo* imaging, the mice were anesthetized i.p. using ketamine (87.5 mg/kg body weight)–xylazine (12.5 mg/kg body weight) cocktail. To minimize the background interference, the fur of the animals was removed using hair clippers and depilatory cream. The anesthetized animals were carefully placed in the imaging cassette, followed by transfer into the chamber in FMT 2500Lx (PerkinElmer Life Sciences). To achieve maximum resolution, the mice were kept in such a position that their lower abdomen was facing the charge-coupled device camera. Scanning was performed using a laser channel of 635 nm. Image processing and analysis were performed using TrueQuant software (PerkinElmer). On the eighth day postimmunization, single-cell suspension of splenocytes and LNs was prepared and stained for CD11c and F4/80 Abs. The samples were acquired in flow cytometry and analyzed for the uptake of E_91_NP_3_-FITC by DCs and macrophages.

### Protection studies

The mice were immunized intranasally using a pipette with E_91_NP3 (E_91_: 10 nM) (three mice/group). After anesthetizing with xylazine (10–12.5 mg/kg i.p.) and ketamine (80–100 mg/kg i.p.), two booster doses with half the initial doses were given intranasally each at a 2-week interval. Two weeks later, mice were aerosol challenged with H37Rv *Mtb* using an inhalation chamber (Glas-Col) to deposit 100 colony-forming units/lung. The mice were sacrificed after 25 days, and lungs were homogenized and plated on 7H11 agar plates containing 10% oleic acid, albumin, dextrose, and catalase. The plates were incubated at 37 °C, and colonies were counted after 25 to 30 days.

### Statistical analysis

Statistical analysis was performed using “one-way analysis of variance with Holm–Sidak’s multiple comparisons test” unless otherwise mentioned by using GraphPad Prism 6 software (GraphPad Software, Inc). “*p* < 0.1” were only considered statistically significant. “∗” *p* < 0.1, “∗∗” *p* < 0.01, “∗∗∗” *p* < 0.001, and “∗∗∗∗” *p* < 0.0001.

## Data availability

Large size datasets are not associated with this study. All raw data for Western blots and qPCR, ELISA, FACS, or microscopic images can be provided upon request to the corresponding author.

## Supporting information

This article contains supporting information.

## Conflict of interest

The authors declare that they have no conflicts of interest with the contents of this article.

## Figures and Tables

**Supplmental Figure 1 A,B:**
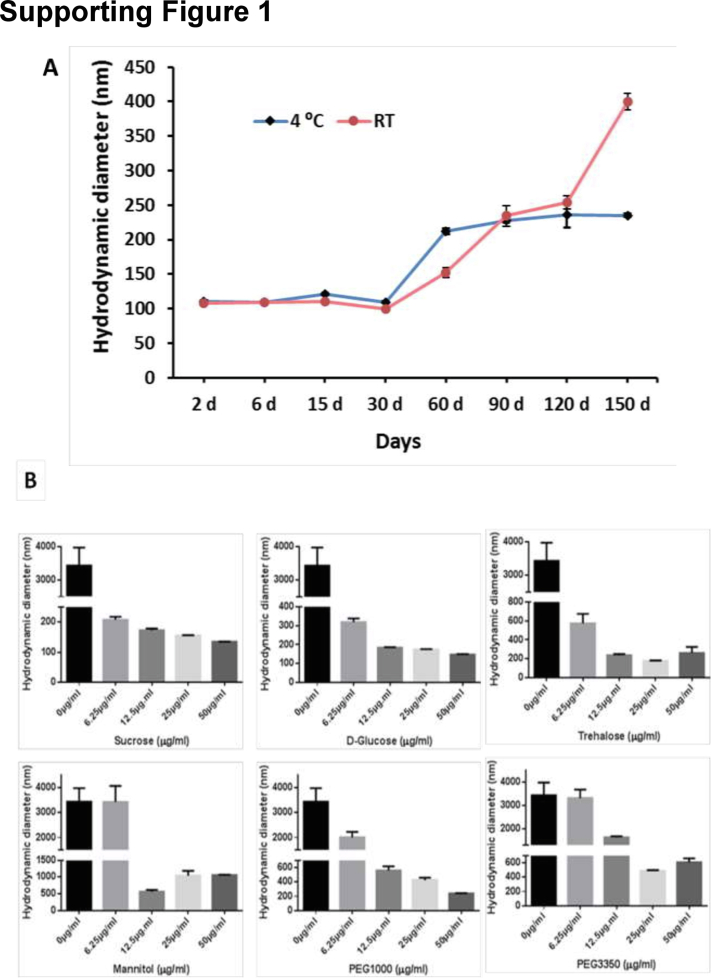


**Supplmental Figure 2 A,B:**
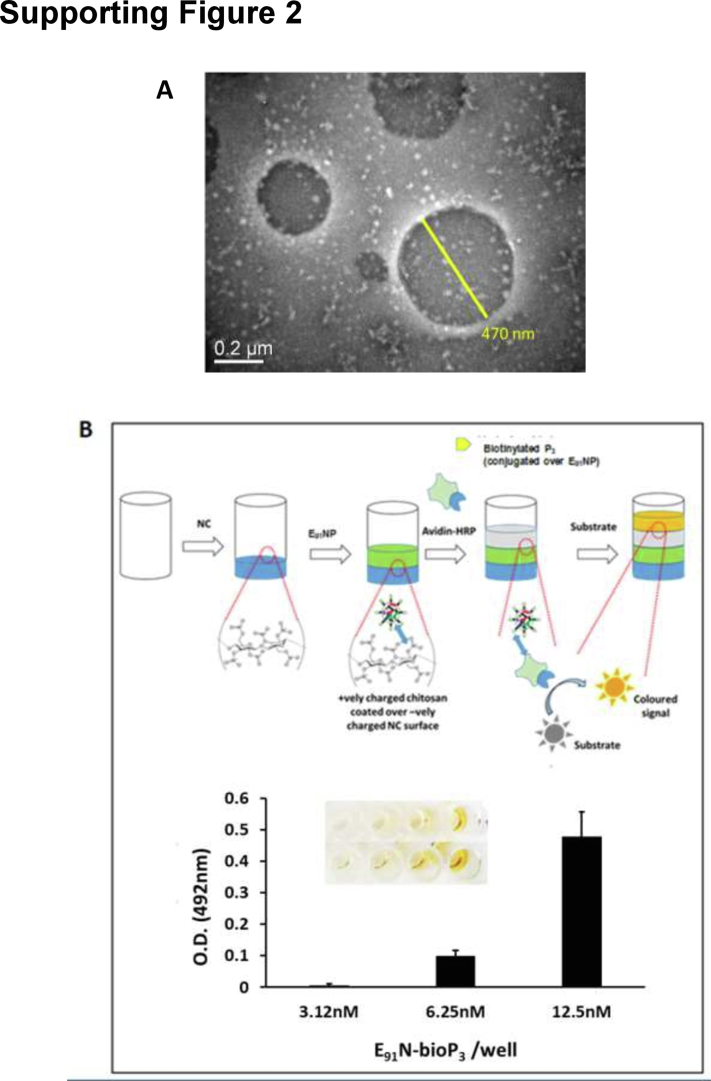


**Supplmental Figure 2 C,D:**
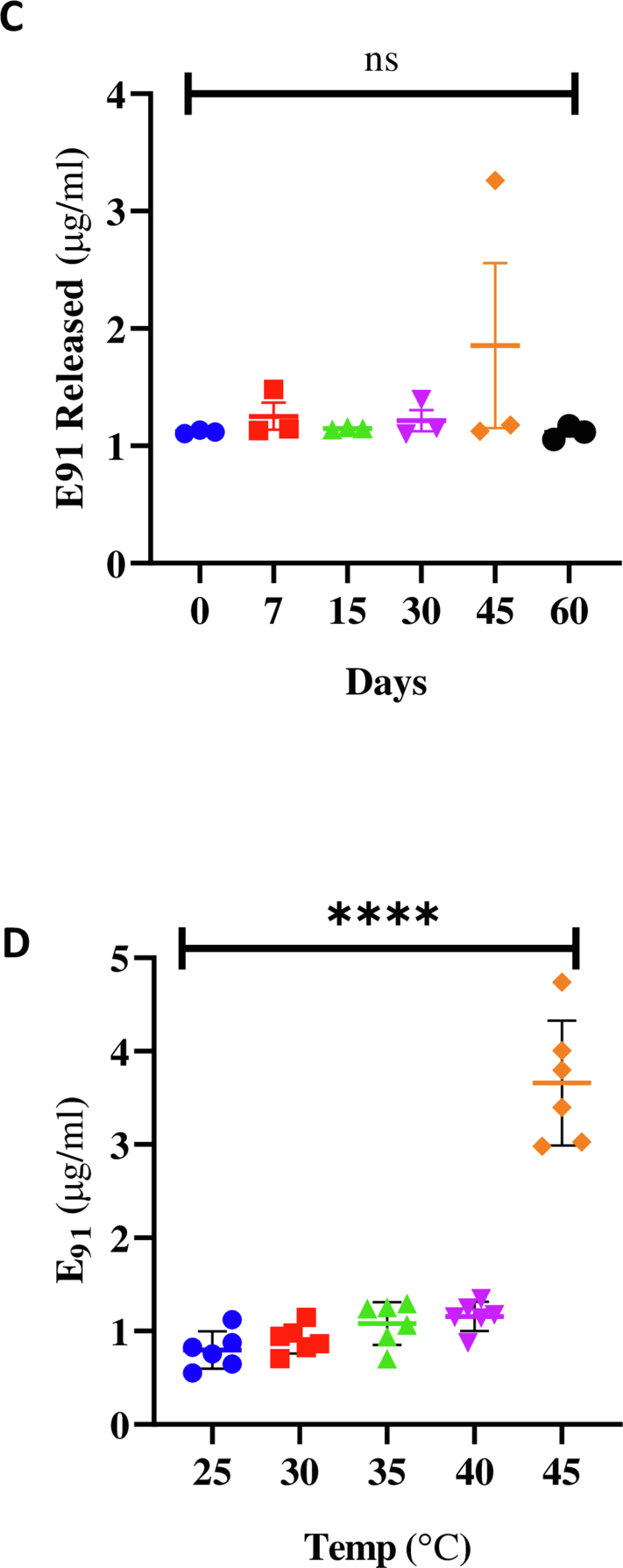


**Supplmental Figure 3 A,B:**
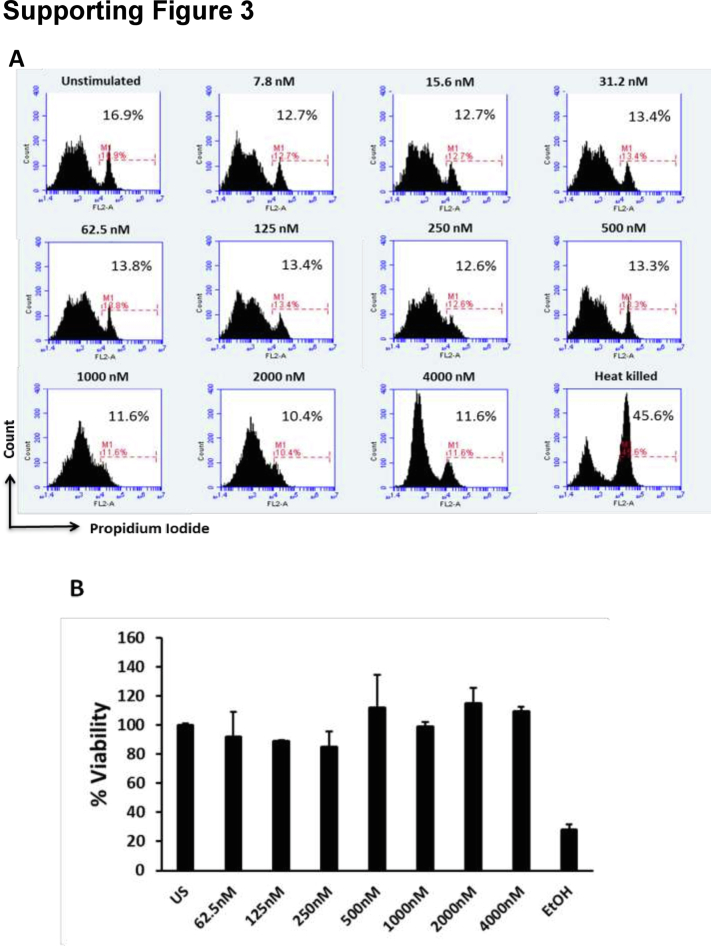


**Supplemental Figure 5B:**
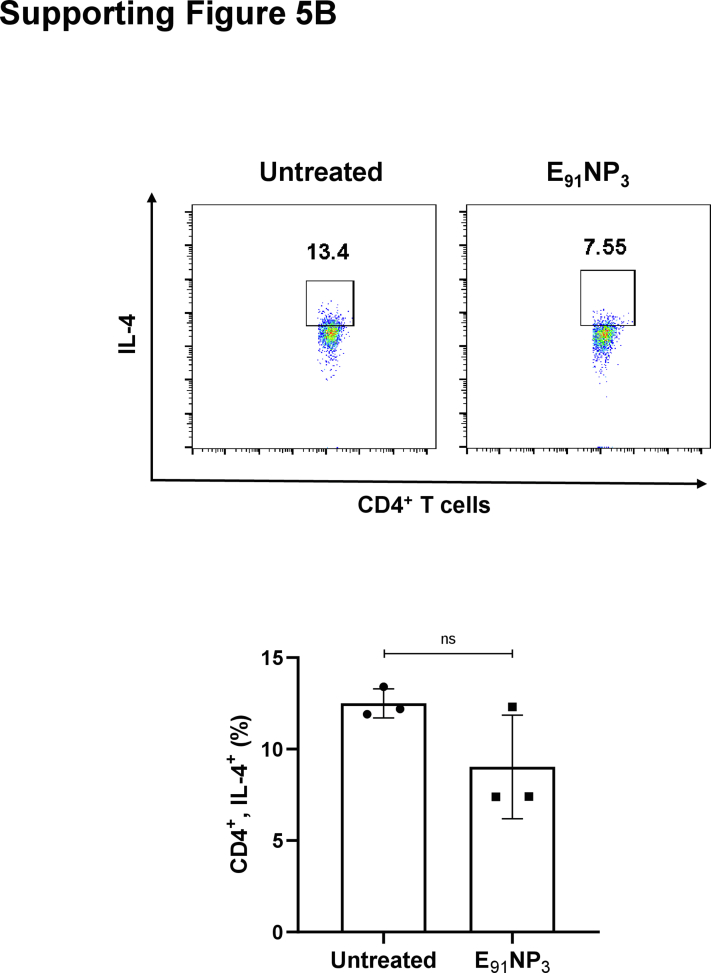


**Supplemental Figure 5C:**
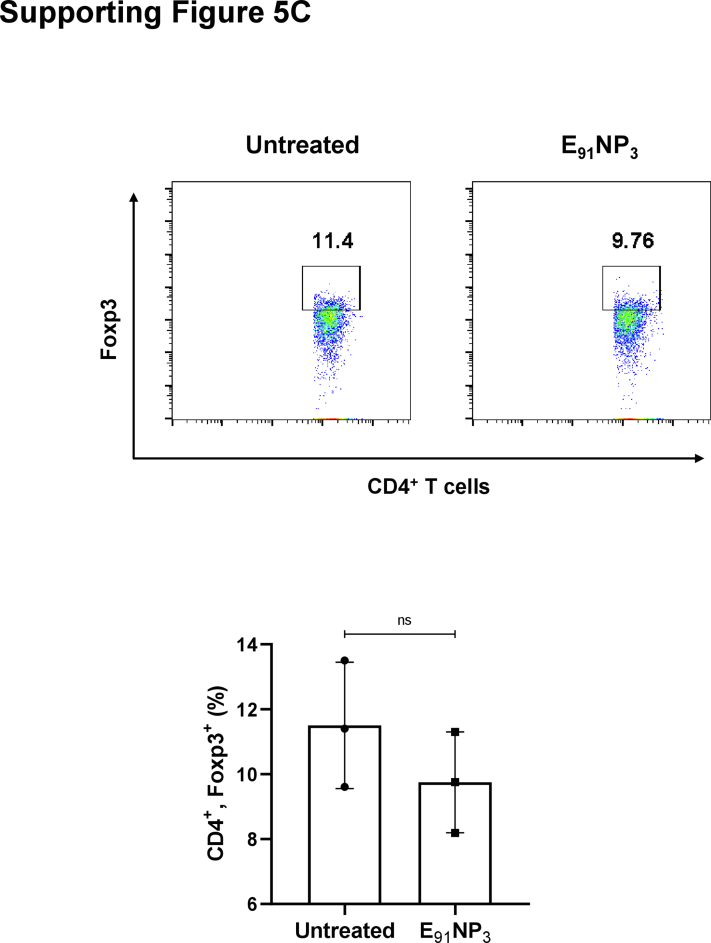


## Supporting information

Supplmental Table 1A

Supplmental Table 1B

Figure S5A
