# Supplementary material for: Targeting dendritic cells with TLR-2 ligand–coated nanoparticles loaded with Mycobacterium tuberculosis epitope induce antituberculosis immunity
Source: J Biol Chem. 2022 Oct 15;298(12):102596. doi: 10.1016/j.jbc.2022.102596 (PMC9674924; doi:10.1016/j.jbc.2022.102596)
Supplement: Supplmental Table 1A [file mmc1.docx]

**Supporting Table 1A**

| **Peptide Seq.** | **Length**  **(AAs)** | **Sequence** | **Class I Immunogenicity**  **Score** |
| --- | --- | --- | --- |
| **91-110** | 20 | SEFAYGSFVRTVSLPVGADE | 0.16365 |
| **91-99** | 9 | SEFAYGSFV | 0.03445 |
| **92-100** | 9 | EFAYGSFVR | 0.00917 |
| **93-101** | 9 | FAYGSFVRT | 0.04708 |
| **94-102** | 9 | AYGSFVRTV | 0.06375 |
| **95-103** | 9 | YGSFVRTVS | 0.2099 |
| **96-104** | 9 | GSFVRTVSL | 0.10466 |
| **97-105** | 9 | SFVRTVSLP | -0.00396 |
| **98-106** | 9 | FVRTVSLPV | -0.07551 |
| **99-107** | 9 | VRTVSLPVG | -0.10264 |
| **100-108** | 9 | RTVSLPVGA | -0.11967 |
| **101-109** | 9 | TVSLPVGAD | 0.01466 |
| **102-110** | 9 | VSLPVGADE | 0.10332 |
