# Supplementary material for: Targeting dendritic cells with TLR-2 ligand–coated nanoparticles loaded with Mycobacterium tuberculosis epitope induce antituberculosis immunity
Source: J Biol Chem. 2022 Oct 15;298(12):102596. doi: 10.1016/j.jbc.2022.102596 (PMC9674924; doi:10.1016/j.jbc.2022.102596)
Supplement: Supplmental Table 1B [file mmc2.docx]

| **Peptide Seq.** | **Length**  **(AAs)** | **Sequence** | **MHC I alleles** |
| --- | --- | --- | --- |
| **91-110** | 20 | SEFAYGSFVRTVSLPVGADE | HLA-A*24:02 (SB) - AYGSFVRTV  HLA-B*07:02 (SB) - GSFVRTVSL  HLA-B*07:02 (WB) - FVRTVSLPV  HLA-B*08:01 (SB) - GSFVRTVSL  HLA-B*40:01 (SB) - SEFAYGSFV |
| **91-99** | 9 | SEFAYGSFV | HLA-B*40:01 (SB) - SEFAYGSFV |
| **92-100** | 9 | EFAYGSFVR | - |
| **93-101** | 9 | FAYGSFVRT | - |
| **94-102** | 9 | AYGSFVRTV | HLA-A*24:02 (SB) - AYGSFVRTV |
| **95-103** | 9 | YGSFVRTVS | - |
| **96-104** | 9 | GSFVRTVSL | HLA-B*07:02 (WB) - GSFVRTVSL  HLA-B*08:01 (SB) - GSFVRTVSL |
| **97-105** | 9 | SFVRTVSLP | - |
| **98-106** | 9 | FVRTVSLPV | HLA-B*07:02 (WB) - FVRTVSLPV |
| **99-107** | 9 | VRTVSLPVG | - |
| **100-108** | 9 | RTVSLPVGA | - |
| **101-109** | 9 | TVSLPVGAD | - |
| **102-110** | 9 | VSLPVGADE | - |

**Supporting Table 1B**
