# Supplementary figures and images for: Targeting dendritic cells with TLR-2 ligand–coated nanoparticles loaded with Mycobacterium tuberculosis epitope induce antituberculosis immunity
Source: J Biol Chem. 2022 Oct 15;298(12):102596. doi: 10.1016/j.jbc.2022.102596 (PMC9674924; doi:10.1016/j.jbc.2022.102596)

Supporting Figure 5A

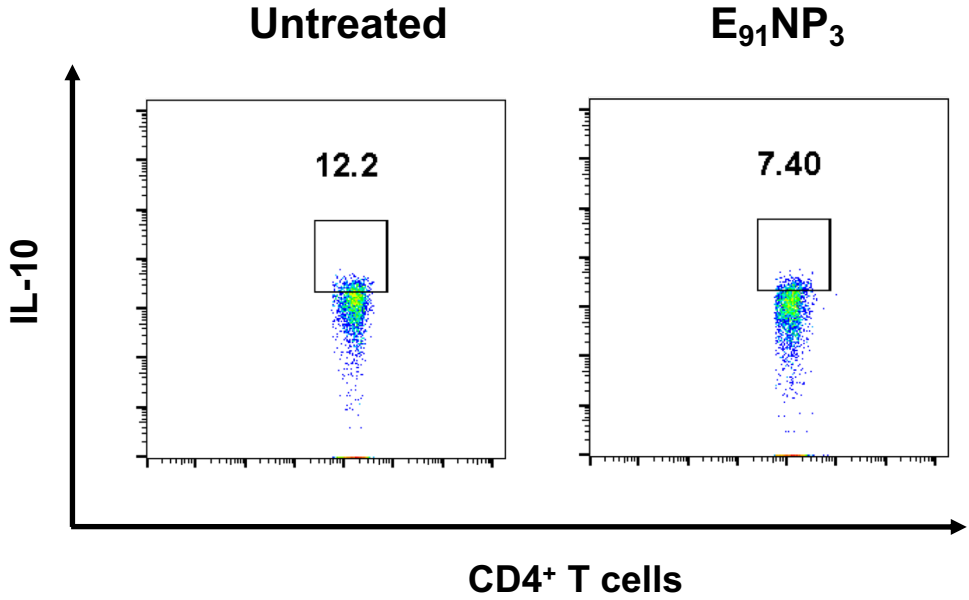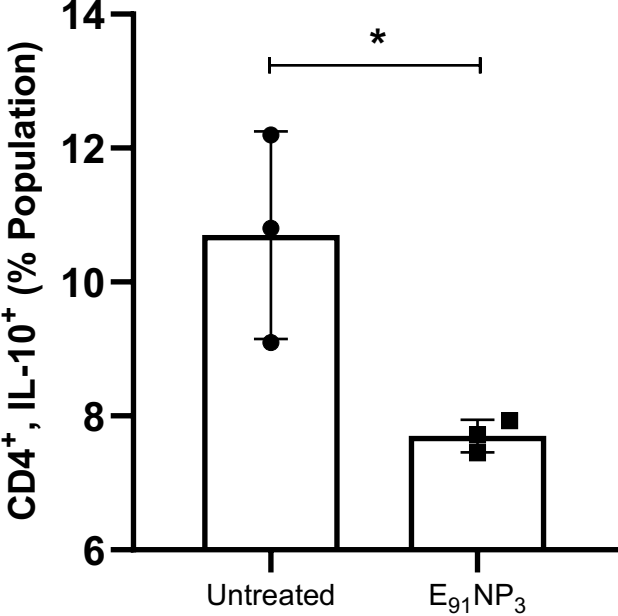

Supplement: Figure S5A [file mmc3.pdf]
